# Hybrid sequencing reveals the genome of a *Chrysochromulina parva* virus and highlight its distinct replication strategy

**DOI:** 10.1186/s12864-025-11700-z

**Published:** 2025-05-17

**Authors:** Delaney Nash, Christine N. Palermo, Ichiro Inamoto, Trevor C. Charles, Jozef I. Nissimov, Steven M. Short

**Affiliations:** 1 Department of Biology, University of Waterloo, Waterloo, ON N2L 3G1 Canada; 2 Department of Biology, University of Toronto Mississauga, Mississauga, ON L5L 1C6 Canada

**Keywords:** Phycodnaviridae, Algavirales, dsDNA virus, Algal viruses, Haptophyte, Prymnesiophyte

## Abstract

*Chrysochromulina parva* (*C. parva*) is a eukaryotic freshwater haptophyte algae found in lakes and rivers worldwide. It is known to be infected by viruses, yet knowledge of the diversity and activity of these viruses is still very limited. Based on sequences of PCR-amplified DNA polymerase B (*polB)* gene fragments, *Chrysochromulina parva* virus BQ1 (CpV-BQ1) was the first known lytic agent of *C. parva*, and was considered a member of the virus family *Phycodnaviridae*, order *Algavirales*. However, the genome of a different *C. parva*-infecting virus (CpV-BQ2, or *Tethysvirus ontarioense*) from another virus family, the *Mesomimiviridae*, order *Imitervirales*, was the first sequenced. Here, we report the complete genome sequence of the putative phycodnavirus CpV-BQ1, accession PQ783904. The complete CpV-BQ1 genome sequence is 165,454 bp with a GC content of 32.32% and it encodes 193 open reading frames. Phylogenetic analyses of several virus hallmark genes including the *polB*, the late gene transcription factor (VLTF-3), and the putative A32-like virion packaging ATPase (Viral A32) all demonstrate that CpV-BQ1 is most closely related to other viruses in the phylum *Megaviricetes* within the order *Algavirales*, family *Phycodnaviridae*.

**Supplementary Information:**

The online version contains supplementary material available at 10.1186/s12864-025-11700-z.

## Introduction

Research into viruses infecting aquatic microbes has seen a sharp increase in recent years. Many aquatic viruses are Nucleocytoplasmic large DNA viruses (NCLDVs), a diverse group of eukaryotic viruses [1] that were recently classified under the viral phylum *Nucleocytoviricota.* The *Nucleocytoviricota* includes the class *Megaviricetes* with three orders and numerous families including the *Mesomimiviridae* and the *Phycodnaviridae* [2]. Viruses belonging to the class *Megaviricetes* are characterized by large double-stranded DNA genomes, typically over 100 kb in size [3], an ability to replicate in either the host’s nucleus [4, 5] or cytoplasm [3, 6], and the presence of a core set of shared genes, including those encoding the major capsid protein, DNA polymerase B, and others [3]. Viruses in the *Megaviricetes* are also notable for their complexity and size, with some members known as “giant viruses” having genomes and virions that rival those of small cellular organisms [6–10]. They infect a wide range of eukaryotic hosts, from algae to animals, and are found in various environments, particularly aquatic ecosystems [7]. They encode genes for DNA replication, transcription, and repair [3], have an extensive genomic plasticity and diversity [3], have acquired genes through lateral gene transfer from various sources [7], and have the potential to significantly alter host metabolism during infection [7].

In addition to their unique genomic characteristics, the *Megaviricetes* play significant ecological roles in various ecosystems, particularly in aquatic environments. They can affect the population dynamics of their unicellular hosts, including controlling algal blooms. For example, *Heterosigma akashiwo* virus (HaV) influences seasonal harmful algal blooms in coastal areas [11]. Moreover, viruses in the *Megaviricetes* contribute to biological carbon export from marine surfaces to deep layers through host-cell death [12, 13]; can impact nitrogen metabolism and fermentation processes in their hosts [14]; and can alter eukaryotic community structures, particularly in marine environments, by infecting various eukaryotic lineages [12, 15–18]. Many of these viruses are involved in metabolic reprogramming by encoding genes involved in nutrient uptake, light harvesting, and central carbon metabolism, allowing them to reprogram host metabolism during infection [7]. The latter includes genes for photosynthesis, diverse substrate transport, and light-driven proton pumps [1]. Indeed, by infecting and lysing their hosts many *Megaviricetes*, such as viruses which infect the massive bloom-forming alga *Gephyrocapsa huxleyi*, drive the release of organic matter into the environment, contributing to nutrient cycling in ecosystems [12, 13, 19–21]. Collectively, these ecological roles highlight the importance of *Megaviricetes* in shaping ecosystem dynamics, particularly in aquatic environments, and their potential impact on global biogeochemical cycles.

One of the most understudied aquatic microbial eukaryotes that is infected by viruses that are likely to be classified within the *Megaviricetes* is *Chrysochromulina parva* (*C. parva*), a freshwater haptophyte alga [22, 23]. It is a small unicellular organism, typically 4–6 μm in size [24] with two flagella (~ 8 μm long) and a long haptonema (up to 10 times the body length) [24]. *C. parva* has a deep groove running the length of the cell, from which the flagella and haptonema emerge, contains two chloroplasts with internal pyrenoids, each associated with a large lipid body, and has a simple cellular morphology with a eukaryotic nucleus, mitochondria with tubular cristae, and a Golgi apparatus. Unlike most *Chrysochromulina* species, *C. parva* lacks visible scales on its cell surface or within the Golgi apparatus [24].

Viruses capable of infecting and lysing *C. parva* were first isolated in 2015 from a Lake Ontario water sample. This sample, collected from the Bay of Quinte, contained a filterable, heat labile, chloroform sensitive lytic agent that was capable of completely lysing cultures of *C. parva* [25]. PCR primers designed to amplify gene fragments from a range of viruses within the *Phycodnaviridae* were used to amplify DNA polymerase B (*polB*) and major capsid protein (MCP) gene fragments [26]. Sanger sequencing of the resulting amplicons led to the identification of a single DNA *polB* gene fragment and 9 unique MCP fragments. Based on the *polB* phylogeny, the lytic agent was named *Chrysochromulina parva* virus BQ1 (CpV-BQ1) and was putatively classified as a phycodnavirus; the presence of multiple MCP fragments allowed the researchers to speculate that more than one type of virus might have been isolated from this water sample [25]. Further culturing, isolation, and high-throughput sequencing experiments led to the assembly of a giant, 437 kb, virus genome encoding 503 open reading frames (ORFs), as well the ~ 23 kb genomes of three polinton-like viruses (PLVs) which are viruses similar to virophages and which need a helper virus for their replication. However, the CpV-BQ1 *polB* gene fragment initially recovered by PCR was not found within this giant virus genome or the PLV genomes, indicating that an additional virus of *C. parva*, named CpV-BQ2, was present in the Bay of Quinte water sample and had been co-cultured along with CpV-BQ1 [27]. Here we report the second genome sequence of a *C. parva* virus noting that this genome assembly was derived from CpV-BQ1 because it encodes an identical *polB* gene as that which originally led to the classification of CpV-BQ1 as a member of the *Phycodnaviridae.* To study this virus’s genome characteristics, we implemented a two-step approach for its sequencing, which combined short read Illumina and long read Nanopore sequencing, coupled with PCR-enabled molecular analysis.

## Results and discussion

### Transmission electron microscopy of CpV-BQ1

**Fig. 1:**
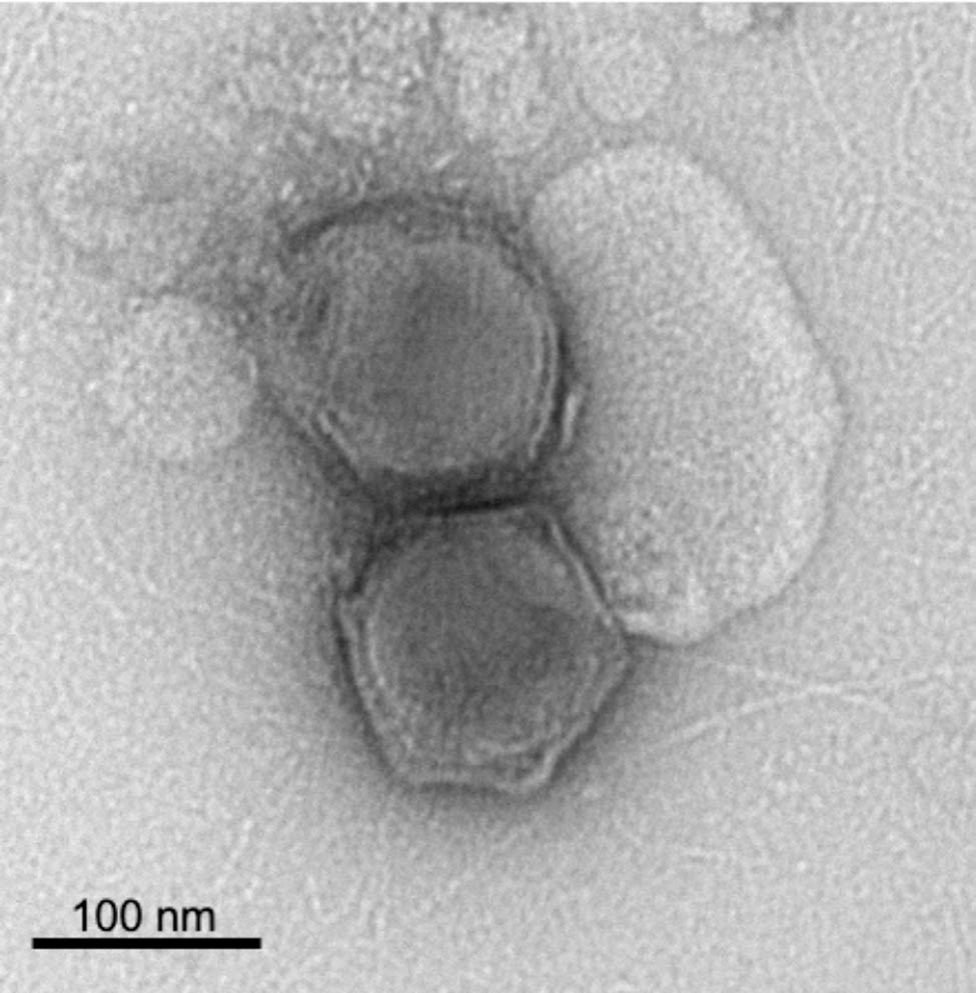
Transmission electron micrograph of negatively stained CpV-BQ1. The scale bar is 100 nm, and the diameter of the bottom particle, when measured diagonally from the top right apex to the bottom left, is 110 nm

CpV-BQ1 virions in *C.parva* lysate were isolated with filtration and concentrated using ultracentrifugation. Then, after negative staining virions were imaged using a transmission electron microscope (TEM). TEM images show the viral capsid is approximately 110 nm when measured from the top right apex to the bottom left apex and has an icosahedral structure (Fig. 1). Viruses within the class *Megaviricetes* exhibit a wide range of capsid sizes, where often larger capsids are associated with bigger genomes [28]. For example, mimivirus has a 400 nm capsid and a 1.2 Mbp genome [8], *Paramecium bursaria chlorella virus* (PBCV-1) has a 190 nm capsid and a 331 kbp genome [29], while *Mantoniella tinhauana virus 1* has a 120.7 nm capsid and a 177,820 bp genome [30]. Due to the CpV-BQ1 small capsid size we also expected a small genome, which was determined to be 165,454 bp after genome assembly (as discussed in the following section).

### Assembly of the CpV-BQ1 genome

A hybrid assembly approach with long Nanopore and short Illumina reads was used to determine the CpV-BQ1 genome. Long read assemblies can produce larger contigs and resolve repetitive genome regions with higher accuracy than short Illumina read assemblies. However, Illumina reads have higher sequence accuracy than long reads and can correct for errors introduced through long read sequencing. Thus, using a combination of long and short reads for assembly produces a genome with higher accuracy and instills greater confidence in the resulting assembly [31, 32]. Hybrid assembly of the CpV-BQ1 genome was performed using the TryCycler pipeline [31]. TryCycler is a robust tool that uses a unique approach to produce assemblies with a high degree of confidence. Most long-read assembly tools work fairly well, however, they are not perfect and can introduce large- and small-scale errors into an assembled genome which often go undetected [31]. To eliminate these issues, TryCycler uses multiple separate long-read assemblies, generated by a variety of assembly tools, as its input and produces a consensus sequence. By using a variety of assembly tools which utilize different approaches/algorithms, bias and/or errors introduced by any one tool can be detected and eliminated [31].

**Fig. 2:**
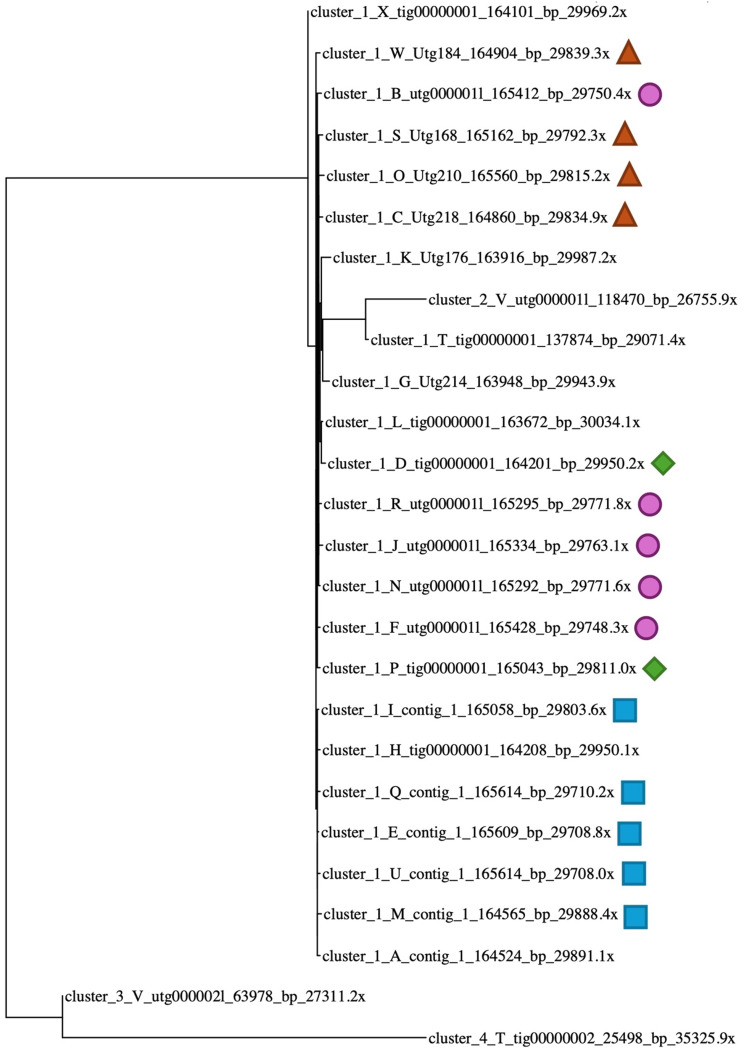
Phylogenetic tree of clustered assembled contigs. The TryCycler clustered assemblies based on the MASH distances between contigs displayed in a phylogenetic tree. Almost all assemblies fall within the cluster 1 branching structure. The 16 assemblies used for reconciling and MSA steps are labeled (e.g., blue squares are Flye assemblies, pink circles are Minipolish assemblies, orange triangles are Raven assemblies, and green diamonds are Canu assemblies)

**Fig. 3:**
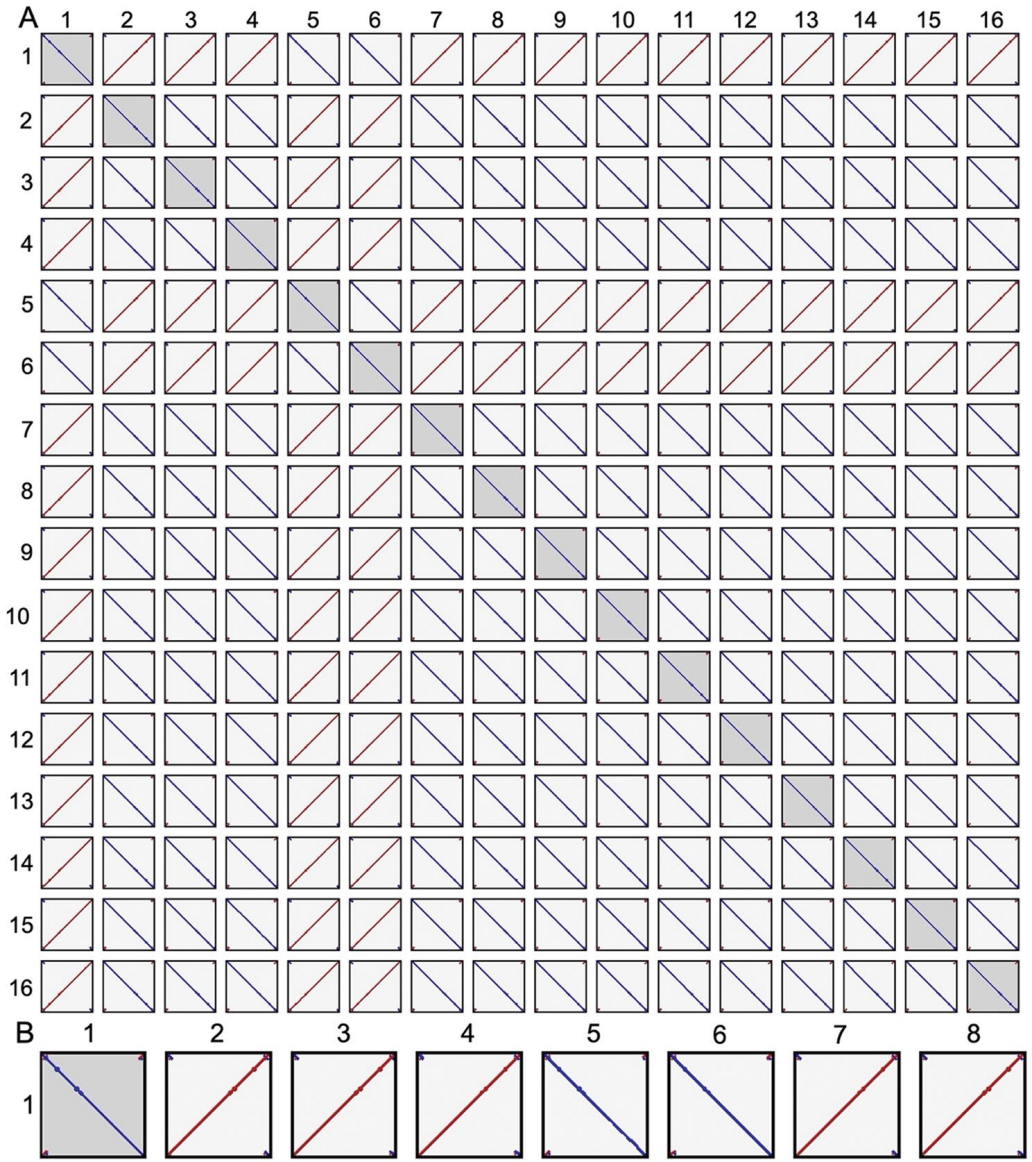
Dotplot Analysis of 16 Assembled Contigs. (**A**) Squares are a visual comparison of all pairwise combinations of the 16 assembled contigs. (**B**) Enlarged dot plots comparing contig one with contigs one through eight, representative plots enable better visualization of the linear relationship between contigs. Solid diagonal blue lines indicate the sequences have the same start and end sites, are highly similar, and do not contain any large gaps or rearrangements. The solid diagonal red lines indicate the sequences are the reverse of one another, are highly similar, and do not contain large gaps or rearrangements

Cleaned and filtered long reads were divided into 24 subsets. Of these, six subsets were assembled with Flye, Minipolish, Raven, and Canu, respectively [33–36]. A total of 26 contigs were assembled of which 23 formed a single phylogenetic cluster (Fig. 2). After reconciling these assemblies, 16 contigs showed high sequence similarity. This included five Flye, and Minipolish assemblies, four Raven assemblies, and two Canu assemblies (Fig. 2). The similarity between the 16 independently generated contigs provided confidence that the assemblies were of very high quality and representative of the true CpV-BQ1 genome. These 16 contigs were used to generate an MSA, reads were partitioned, then a consensus genome was generated and polished with Illumina reads producing a 165,454 bp genome. Additionally, attempts to circularize the genome during reconciling were unsuccessful. Analysis of these contigs using a dotplot shows the same discrete start and end point in all 16 contigs which indicates the genome has a linear topology (Fig. 3). If genomes had a circular topology, we would expect the start and stop sites to vary amongst the 16 assembled genomes, causing the lines within each dotplot to start along the axis edge instead of the corner. Additionally, if some of our assemblies contained gaps or large rearrangements the plots would contain lines with gaps or a discontinuous arrangement. Our dotplot indicates these 16 assemblies are not circular and do not contain gaps or large rearrangements (Fig. 3).

**Fig. 4:**
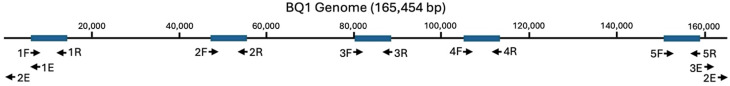
Diagram of CpV-BQ1 genome primer binding sites. The complete 165,454 bp CpV-BQ1 genome with the primer binding sites used for genome validation. Primer binding sites are represented by arrows specific to the direction of amplification. Primer pairs 1F&1R, 2F&2R, 3F&3R, 4F&4R, and 5F&5R used to amplify 9,000 bp regions in the genome indicated by blue rectangles. Primer pairs 1E&2E, 1E&3E, 2E&2E, and 2E&3E were used to PCR amplify across the genome ends (Supplementary Table 1)

To validate the assembled sequence PCR amplifications, Nanopore sequencing and TryCycler assembly were performed to recover and verify the sequence of five 9,000 bp regions within the genome (Fig. 4). Additionally, to verify the genomes linear topology, four PCR amplifications across the genome ends were attempted (Fig. 4).

The five ~ 9,000 bp regions were easily PCR amplified, sequenced, and assembled with TryCycler (Supplementary Table 2). Alignment of the assembled contigs to the CpV-BQ1 genome showed almost 100% sequence identity to their respective sites. The very ends of assembled PCR regions # two and # five contained one and 17 bases that differ from the CpV-BQ1 genome, respectively, which can be attributed to low read coverage in these areas. Overall, the similarity of PCR amplified sequences to the assembled genome provides further confidence in the assembly accuracy. Attempts to amplify genome ends to test for circularization were unsuccessful. Of these four attempted PCR amplifications (Fig. 4), nanopore reads were obtained from three reactions. However, attempts at read assembly with TryCycler resulted in clusters with many spurious, incomplete, and misassembled contigs. Thus, the inability to amplify sequences which span the genome ends provides additional confirmation that the CpV-BQ1 genome has a linear topology.

### Characterization and description of the CpV-BQ1 genome

**Fig. 5:**
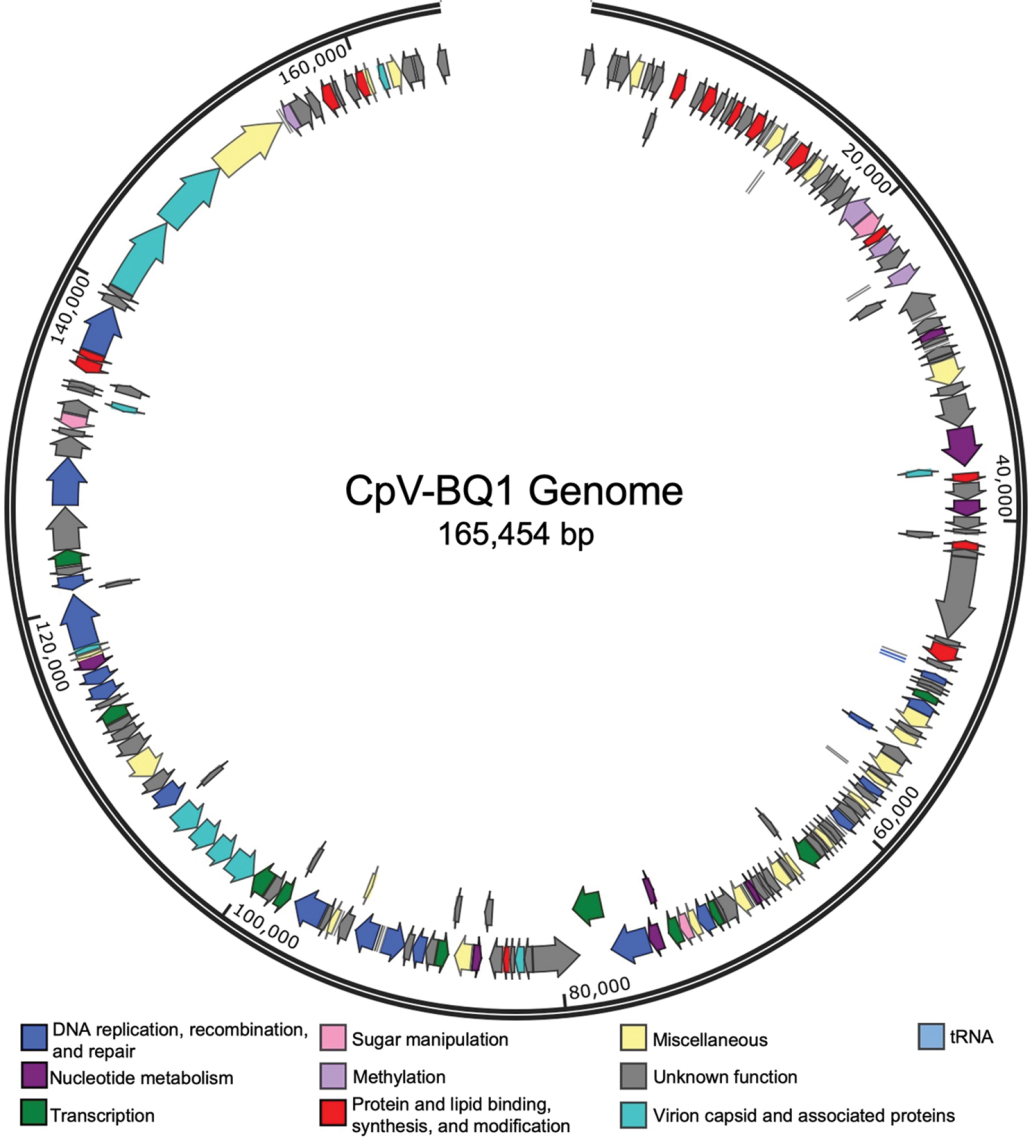
ORF Predictions in the CpV-BQ1 Genome. ORFs represented by arrows are aligned to the 165,454 bp CpV-BQ1 genome oriented in their coding direction. Arrows are coloured based on their hypothesized functional group. Blue arrows for DNA replication, recombination, and repair; dark purple for nucleotide metabolism and DNA packaging; green for transcription; pink for sugar manipulation; lavender for methylation; red for protein and lipid binding, synthesis, and modification; aqua for virion capsid and associated proteins; yellow for miscellaneous proteins; light blue for tRNA; and grey for all proteins with unknown functions. The genome is presented as circular for presentation purposes only. Nucleotide positions are denoted at every 20,000 bps

Assembly of the CpV-BQ1 genome sequence produced a linear genome of 165,454 bp with a GC content of 32.32%. Three transfer RNA (tRNA) sequences and 193 coding sequences (CDSs) were identified in the genome which ranged from 44 to 1693 amino acids (aa) in length, with an average size of 260 aa (Fig. 5, Supplementary Table 3). Coding sequences were found in slightly higher prevalence on the positive strand than the negative strand, occurring at a rate of 58.5% and 41.5%, respectively. Of the 193 identified CDSs, 92 (47.67%) were assigned putative functions based on homology to known genes and protein domains, while the remaining 101 (52.33%) could not be assigned functions and were designated as hypothetical proteins (Supplementary Table 3). Functionally annotated CDSs were placed into eight general functional groups (Fig. 5; Table 1). A total of 19 CDSs were involved in DNA replication, recombination, and repair; eight in nucleotide metabolism and DNA packaging; 10 in transcription; three in sugar manipulation; four in DNA methylation; 15 in protein and lipid binding, synthesis, and modification; 12 in virion capsid and associated proteins, and 21 encoded miscellaneous functions (Table 1).

**Table 1:** List of all characterised genes in the CpV-BQ1 genome

Protein	Locus	Accession	Start	End
**DNA and RNA replication, recombination, and repair**				
yqaJ-like viral recombinase domain containing like-protein	BQ1_063	XNN68166	49,504	49,701
pyrimidine dimer DNA glycosylase, endonuclease V	BQ1_064	XNN68167	50,107	49,682
DNA polymerase family X	BQ1_068	XNN68171	52,187	51,462
GxxExxY protein	BQ1_072	XNN68175	54,374	54,754
Holliday junction resolvase, A22	BQ1_078	XNN68181	57,463	57,972
SWIB/MDM2-domain containing protein	BQ1_083	XNN68186	60,100	60,714
DNA topoisomerase I	BQ1_104	XNN68207	70,386	71,225
ATP-dependent DNA ligase	BQ1_110	XNN68213	74,487	76,832
proliferating cell nuclear antigen, PCNA	BQ1_124	XNN68227	88,347	89,114
VV A18-like helicase	BQ1_126	XNN68229	90,943	89,633
NTPase/helicase	BQ1_128	XNN68231	91,438	92,781
P-loop containing nucleoside triphosphate hydrolase	BQ1_134	XNN68237	94,988	96,880
putative DNA primase/helicase	BQ1_144	XNN68247	107,635	106,412
Flap endonuclease 1	BQ1_152	XNN68255	115,062	114,115
YqaJ-like viral recombinase domain containing protein	BQ1_153	XNN68256	116,022	115,118
DNA topoisomerase II	BQ1_157	XNN68260	117,625	120,840
exodeoxyribonuclease III	BQ1_159	XNN68262	121,988	121,146
Helicase	BQ1_163	XNN68266	126,370	129,303
DNA polymerase type-B	BQ1_174	XNN68277	135,953	138,937
**Nucleotide transport and metabolism**				
thymidine kinase	BQ1_040	XNN68143	28,623	28,096
ribonucleoside-diphosphate reductase large subunit	BQ1_048	XNN68151	34,374	36,686
ribonuclease-diphosphate reductase small subunit	BQ1_052	XNN68155	38,805	39,767
deoxycytidylate deaminase	BQ1_098	XNN68201	67,554	67,210
deoxyuridine 5’-triphosphate nucleotidohydrolase	BQ1_108	XNN68211	73,618	73,169
thymidylate synthase ThyX	BQ1_109	XNN68212	73,681	74,451
NUDIX hydrolase	BQ1_119	XNN68222	85,372	84,851
Viral A32 protein	BQ1_154	XNN68257	116,887	116,066
**Transcription**				
RNA cap guanine-N2 methyltransferase family protein	BQ1_067	XNN68170	51,398	50,886
transcription factor IIB	BQ1_090	XNN68193	62,855	63,874
transcription elongation factor S-II	BQ1_103	XNN68206	69,838	70,341
ribonuclease III	BQ1_107	XNN68210	72,453	73,172
mRNA-capping enzyme	BQ1_111	XNN68214	76,891	78,837
MYM-type Zinc finger with FCS sequence motif-containing protein	BQ1_122	XNN68225	87,611	86,892
TATA-box binding protein	BQ1_136	XNN68239	97,984	97,247
late transcription factor VLTF3	BQ1_138	XNN68241	98,765	99,838
mRNA-capping enzyme	BQ1_150	XNN68253	112,713	113,603
transcription initiation factor TFIIIB	BQ1_161	XNN68264	122,685	123,626
**Sugar manipulation**				
glycosyltransferase family 2 protein	BQ1_030	XNN68133	20,111	21,163
glycosyltransferase 17	BQ1_106	XNN68209	71,804	72,418
glycosyltransferase	BQ1_166	XNN68269	131,902	131,078
**DNA Methylation**				
DNA cytosine-5 methyltransferase	BQ1_029	XNN68132	20,038	18,737
FkbM family methyltransferase protein	BQ1_032	XNN68135	21,810	22,706
D12 class N6 adenine-specific DNA methyltransferase protein	BQ1_035	XNN68138	23,949	24,851
SAM-dependent methyltransferase	BQ1_181	XNN68284	155,423	154,812
**Protein and lipid binding**,** synthesis**,** and modifications**				
F-box domain containing protein	BQ1_008	XNN68111	5793	6443
F-box domain containing protein	BQ1_010	XNN68113	7749	8501
F-box domain containing protein	BQ1_013	XNN68116	9687	10,196
F-box domain containing protein	BQ1_015	XNN68118	11,116	11,862
RING finger E3 ubiquitin protein ligase	BQ1_022	XNN68125	14,225	15,103
E3 ubiquitin-protein ligase	BQ1_031	XNN68134	21,217	21,729
translation initiation factor 4E	BQ1_050	XNN68153	37,156	37,707
Putative Metallopeptidase WLM domain containing protein	BQ1_056	XNN68159	41,801	41,286
DNAJ homolog subfamily A member	BQ1_060	XNN68163	47,801	48,868
E3 ubiquitin-protein ligase	BQ1_092	XNN68195	64,318	64,644
N1R/p28-like protein	BQ1_116	XNN68219	83,104	83,520
patatin-like phospholipase family protein	BQ1_172	XNN68275	135,387	134,623
WLM domain-containing protein	BQ1_173	XNN68276	135,893	135,411
F-box domain containing protein	BQ1_184	XNN68287	158,155	157,388
F-box domain containing protein	BQ1_187	XNN68290	160,285	159,638
**Virion Capsid and Associated proteins**				
ERV/ALR sulfhydryl oxidase protein	BQ1_049	XNN68152	37,107	36,658
Ac78 protein	BQ1_099	XNN68202	67,798	67,643
minor capsid P9 transmembrane helices containing protein	BQ1_114	XNN68217	82,228	82,785
putative major capsid protein A	BQ1_139	XNN68242	101,585	99,924
putative major capsid protein B	BQ1_140	XNN68243	102,955	101,687
putative major capsid protein C	BQ1_141	XNN68244	104,372	103,038
putative major capsid protein D	BQ1_142	XNN68245	106,012	104,462
Thioredoxin	BQ1_156	XNN68259	117,244	117,561
Glutaredoxin-like protein	BQ1_168	XNN68271	133,267	132,848
tail fiber protein containing WIAG-tail domain	BQ1_177	XNN68280	140,154	145,088
fibronectin type III domain containing tail fiber protein	BQ1_178	XNN68281	145,167	149,783
tape measure protein	BQ1_189	XNN68292	161,545	161,105
**Miscellaneous**				
RNA binding domain containing protein	BQ1_004	XNN68107	3644	2958
transmembrane protein similar to AMEV204	BQ1_018	XNN68121	12,505	13,218
Zinc-finger, ring type domain contaning protein	BQ1_024	XNN68127	15,552	16,211
Diverse functionality domain-containing protein	BQ1_045	XNN68148	30,081	31,643
AAA family ATPase/CfxQ-like protein	BQ1_069	XNN68172	52,224	53,210
DUF5871 protein	BQ1_071	XNN68174	53,711	54,340
DUF5764 protein	BQ1_074	XNN68177	55,548	56,522
DUF5759 protein	BQ1_076	XNN68179	56,932	57,294
DUF5754 protein	BQ1_080	XNN68183	58,923	58,603
putative Mn2+ efflux pump MntP	BQ1_087	XNN68190	62,104	61,712
DUF5871 protein	BQ1_094	XNN68197	65,085	65,804
2OG-Fe(II) oxygenase	BQ1_100	XNN68203	67,910	68,626
DUF5872 protein	BQ1_105	XNN68208	71,244	71,759
DUF5767 protein	BQ1_120	XNN68223	85,474	86,505
DUF1599 protein	BQ1_129	XNN68232	93,038	92,778
DUF5760 protein	BQ1_132	XNN68235	94,057	94,470
IQ motif containing protein	BQ1_146	XNN68249	110,246	108,597
type II secretory pathway component PulF	BQ1_155	XNN68258	117,159	116,935
fibronectin type III domain containing transmembrane protein	BQ1_179	XNN68282	149,818	154,509
high mobility group protein	BQ1_188	XNN68291	160,585	160,349
RNA binding domain containing protein	BQ1_190	XNN68293	161,786	162,472

All genes in the CpV-BQ1 genome with an identifiable function or domain architecture. Gene name, locus, as well as start and end position within the CpV-BQ1 genome are provided. These protein encoding genes are divided into functional groups including, DNA replication, recombination, and repair; nucleotide metabolism and DNA packaging; transcription; sugar manipulation; DNA methylation; protein and lipid binding, synthesis, and modifications; virion capsid and associated proteins; and miscellaneous

### Taxonomy

**Fig. 6:**
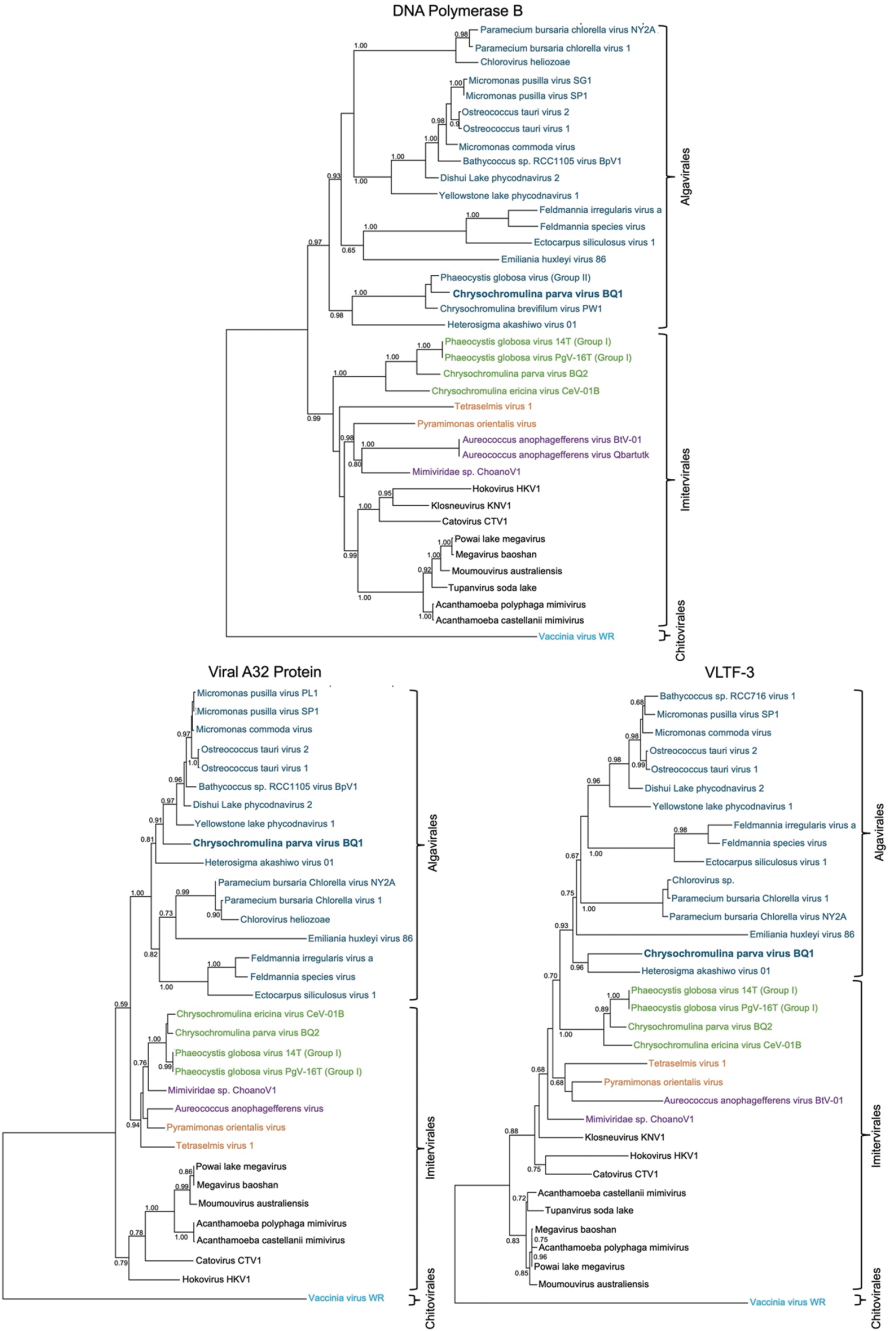
Phylogenetic analysis of three CpV-BQ1 genes universally found in NCLDV genomes. Phylogenetic trees generated to determine the taxonomy of the CpV-BQ1 virus using the Phylogenetic maximum likelihood (PhyML) algorithm in Seaview v5.0. Trees were generated using the protein sequences for the universal NCLDV genes *polB*, viral A32 protein, and VLTF-3 (Supplementary Table 4). Protein sequences are from viruses belonging to either the *Algavirales*, *Imitevirales*, or *Chitovirales* order. Families within each order are colour coded, *Phycodnaviridae* in blue, *Mesomimiviridae* in green, *Schizomimiviridae* in purple, *Allomimivirdae* in orange, *Mimiviridae* in black, and *Poxviridae* in light blue

Viruses within the *Megaviricetes* (previously and informally known as NCLDVs) share a few common characteristics, including the presence of a dsDNA genome typically over 100 kb, which encodes a handful of universal NCLDV genes [3]. These genes include the major capsid protein (MCP), a DNA polymerase B (*polB*), the viral A32-like packing ATPase, and the viral late transcription factor 3 (VLTF-3) [3]. Additionally, although the D5-helicase is considered a universal NCLDV gene, it is not found in viruses in the *Phycodnaviridae* family, nor in the CpV-BQ1 genome [3]. Of the universal genes, the MCPs share little sequence identity, and thus cannot be used reliably for phylogenetic analysis. Phylogenetic analysis of the CpV-BQ1 genome was therefore performed using *polB*, viral A32-like packing ATPase, and VLTF-3 (Fig. 6). In all three phylogenetic trees, the CpV-BQ1 groups closely with viruses in the *Phycodnaviridae* family (Fig. 6) such as the Heterosigma akashiwo virus 01, a member of the *Phycodnaviridae* family and the single member of the genus *Raphidovirus*. Within the DNA polymerase B phylogenetic tree CpV-BQ1 groups closest to two viruses within the genus *Prymnesiovirus* [18], Chrysochromulina brevifilum virus PW1 (CbV-PW1) [37] and Phaeocystis globosa virus 08T (PgV-08T) [38]. Molecular analysis of the A32 ATPase and VLTF-3 genes in CbV-PW1 and PgV-08T has not been performed, thus we could not include them in our phylogenetic analysis. Nonetheless, based on the similarity of the *polB* gene and the fact these three viruses all have prymnesiophyte hosts [18] suggests the most appropriate classification for CpV-BQ1 is within the *Prymnesiovirus* genus. Thus, our phylogenetic analysis supports the inclusion of CpV-BQ1 in the NCLDV *Phycodnaviridae* family and suggests it can most appropriately be placed within the *Prymnesiovirus* genus.

### DNA and RNA replication, recombination, and repair enzymes

NCLDVs encode a suite of genes which enable the replication of their own genome with little reliance on their host’s genome replication machinery [39, 40]. A total of 19 proteins involved in DNA/RNA replication, recombination, and repair were identified in the CpV-BQ1 genome. This consort of enzymes enables CpV-BQ1 to facilitate much of its own DNA replication processes. These replication associated genes include a DNA polymerase type-B (*polB*), a DNA polymerase family X protein, an A22 Holliday junction resolvase, a proliferating cell nuclear antigen sliding clamp (PCNA), topoisomerase I and II, four nuclease proteins, four helicase proteins, two YqaJ-like viral recombinases, an ATP-dependent DNA-ligase, a P-loop nucleoside triphosphate hydrolase, and a SWIIB/MDM2-domain protein [39, 40]. Some NCLDVs, such as poxviruses [39], mimiviruses [5, 6], and phycodnaviruses [4, 29] replicate and package their genomes within the cytoplasm of their host cell in subcellular compartments surrounded by endoplasmic reticulum membranes, which are referred to as virus factories [5, 39]. However, other NCLDVs, such as members of the family *Phycodnaviridae*, replicate their genome within the nucleus of their host, then package virions in the cytoplasmic virus factories [4, 5]. To explore the location of CpV-BQ1 genome replication, proteins involved in DNA replication were analyzed for the presence of nuclear localization signals (NLS) using DeepLoc 2.1 [41]. Indeed, NLS signals were identified in 12 of the 19 predicted DNA replication, recombination, and repair enzymes, including the DNA-dependent DNA replication polymerase, *polB*. This suggests that at least some part of the DNA replication process occurs within the host cell nucleus.

### Transcription

Most NCLDVs encode a DNA-dependent RNA polymerase which allows them to transcribe their viral mRNA within the cytoplasm [42]. However, some members of the *Phycodnaviridae* family, such as chloroviruses and prasinoviruses, do not encode an RNA polymerase gene. Instead, they rely on the host’s RNA polymerase and enter the nucleus to initiate viral gene transcription [43]. Similarly, the CpV-BQ1 genome does not encode a recognizable RNA polymerase and therefore its lytic cycle most likely involves a nuclear localization step necessary for gene transcription.

To gain control of the host RNA polymerase II (RNA pol), CpV-BQ1 encodes several eukaryotic-like transcription factors (TFs) which likely interact with the host’s RNA pol and alter its activity. CpV-BQ1 encodes two transcription factor IIB (TFIIB) proteins and a TATA box binding protein (TBP), which are similar to general eukaryotic TFs [44]. During eukaryotic transcription initiation, RNA pol is recruited to the DNA promoter by a complex of general TFs including TFIIB and TBP. With virus encoded TFs, viruses can redirect the RNA pol to initiate the transcription of viral genes [44]. CpV-BQ1 also encodes two specific TFs which activate viral genes expressed in the late transcription phase, including virus late transcription factor 3 (VLT-3) and an MYM-type zinc-finger FCS motif containing protein [45–47].

A handful of other genes important for transcription are encoded by the CpV-BQ1 genome. Transcription elongation factor S-II (TFIIS) stimulates the cleavage activity of RNA pol when the transcription complex becomes stalled, allowing transcription to be restarted at the newly created 3’ prime end [48]. Two mRNA-capping enzymes are encoded which ensure the protection and efficient transcription of mRNA. A protein Blast analysis of these two mRNA-capping enzymes show they may differ in their evolutionary origin and possibly function. The first is encoded at locus BQ1_111 and is similar to bacterial and eukaryotic mRNA capping enzymes, while the second locus is similar to archaeal and viral mRNA capping enzymes. Moreover, an RNA cap guanine-N2 methyltransferase family protein likely methylates the 5’ prime cap on mRNA, which is suspected to increase the synthesis of viral transcripts [49]. Lastly, the enzyme ribonuclease III (RNase III) is encoded by the CpV-BQ1 genome. RNase III proteins are involved in the processing and maturation of RNA species, including tRNA, rRNA, and mRNA, as well as the degradation of mRNAs. The specific role of RNase III encoded by NCLDVs is not well understood, however it is suspected that it is involved in RNA cleavage and tRNA maturation processes [50].

During the nuclear infection stage, chloroviruses such as Paramecium bursaria chlorella virus (PBCV-1) not only redirect host transcriptional machinery, but they also repress the expression of host genes [51]. It is likely that CpV-BQ1 uses mechanisms similar to PBCV-1 to decrease the transcription of host genes and prioritize the expression of its own genes. In the virion, PBCV-1 packages methylation, restriction endonuclease, and chromatin-remodeling enzymes which are released upon nuclear entry of virion particles. In consort, these enzymes methylate, degrade, and remodel host DNA effectively downregulating the production of host transcripts [51]. CpV-BQ1 encodes several methylase enzymes and endonucleases, some of which may target host DNA. Additionally, CpV-BQ1 encodes a SWIB/MDM2-domain containing protein; the SWIB domain, although not fully understood, can function as a transcriptional activator or as a chromatin-remodeling protein [52]. Thus, circumstantial evidence suggests the CpV-BQ1 genome contains the necessary protein apparatus to downregulate host gene transcription.

### Nucleotide transport and metabolism proteins

**Fig. 7:**
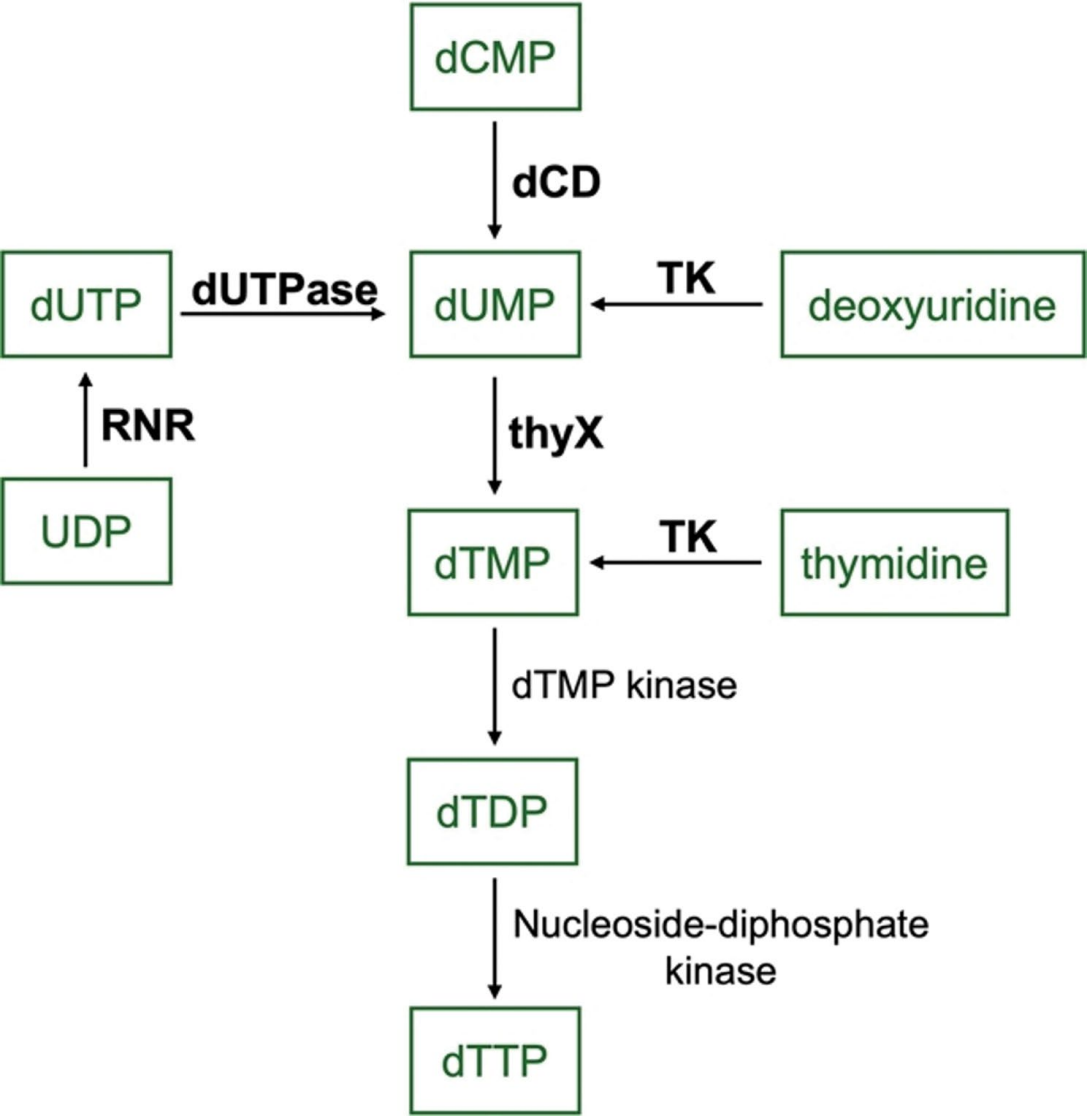
Biosynthesis of deoxythymidine triphosphate (dTTP). KEGG ontology pyrimidine metabolism pathway (https://www.kegg.jp/pathway/ec00240+3.5.4.12). Substrates are in green boxes, enzymes written in black text convert substrates following the direction of the arrow heads. Enzyme names that are bolded are encoded by the CpV-BQ1 genome, while the remaining enzymes are not encoded by the CpV-BQ1 genome.

To facilitate rapid genome replication, some NCLDVs encode DNA precursor metabolism proteins which generate pools of available deoxythymidine triphosphate (dTTP) [39]. The CpV-BQ1 genome encodes several genes which are important for dTTP synthesis, including deoxycytidylate deaminase (dCD), thymidine kinase (TK), deoxyuridine 5’-triphosphate nucleotidohydrolase (dUTPase), thymidylate synthase ThyX (thyX) and ribonucleoside-diphosphate reductase (RNR) (Fig. 7) [53].

The three enzymes dCD, TK, and dUTPase convert their respective substrates to dUMP, an essential precursor molecule for the production of dTMP by the enzyme thyX (Fig. 7). Furthermore, TK can also phosphorylate available thymidine to produce dTMP [54]. dTMP can then be phosphorylated by dTMP kinase then nucleoside diphosphate kinase to produce dTTP which can be incorporated into replicating viral DNA, however, the CpV-BQ1 genome does not encode either of these enzymes (Fig. 7). In addition to contributing to dTTP biosynthesis, RNR can also convert CDP to dCDP with help from the protein glutaredoxin, which is encoded by the CpV-BQ1 genome. dCDP can then be phosphorylated by nucleoside-diphosphate kinase to produce dCTP which can be used for DNA synthesis. However, it has been shown that these proteins are only essential for maintaining dCTP levels when similar host enzymes have been depleted [55]. Other studies have demonstrated that glutaredoxin is an important component in a redox cascade reaction that is required for virion assembly [56]. Analysis of the CpV-BQ1 encoded glutaredoxin with subcellular prediction tool DeepLoc identified an endoplasmic reticulum (ER) localization signal, thus this protein is more likely to be located at ER virus factories and involved in virion assembly and packaging.

The CpV-BQ1 genome also encodes an NUDIX hydrolase. NUDIX proteins hydrolyze substrates containing a nucleoside diphosphate linked to some other moiety which increases free nucleotides available for metabolism [57]. It remains unclear which substrate(s) the CpV-BQ1 encoded NUDIX hydrolase has specificity to, however it has been shown that NUDIX enzymes in NCLDVs Vaccinia Virus, African Swine Fever Virus (ASFV), and Mimivirus L375 function as mRNA decapping enzymes. This decapping process is thought to facilitate mRNA turnover and increase the availability of dNTPs [58].

### DNA methylation

Methyltransferases (MTases) are present in all domains of life however they are not highly prevalent amongst viruses. Within viruses, MTases are most often reported in bacteriophages and some members of the *Phycodnaviridae* family [53, 59]. Phycodnaviruses often use their encoded MTases to methylate their own genome. Moreover, it has been observed that some chloroviruses encode their own restriction-modification (R-M) systems, complete with type II site specific restriction enzymes to degrade host or foreign DNA and MTases to protect their own genome from degradation [59, 60]. The CpV-BQ1 genome encodes five methyltransferase enzymes, including a D12 class N6 adenine-specific DNA methyltransferase protein (mA6-MTase), DNA cytosine-5 methyltransferase (mC5-MTase), FbkM methyltransferase, a SAM-dependent methyltransferase, and the previously discussed RNA cap guanine-N2 methyltransferase. The possibility of a CpV-BQ1 encoded R-M system was investigated but is unlikely as we could not identify any type II site-specific restriction enzymes in the CpV-BQ1 genome.

mA6-MTase methylates the amino group at the C6 position of adenine in specific nucleotide motifs, creating an N^6^-methyl-adenine modification [59, 61]. N^6^-methyl-adenine modifications are rare amongst eukaryotes but are widespread amongst *Phycodnaviridae* family members [54, 59, 62, 63]. The CpV-BQ1 genome is AT-rich, with a 67.68% AT content, which makes adenine a highly prevalent nucleotide and robust candidate for methylation. Therefore, this enzyme likely specifically methylates the CpV-BQ1 genome to provide protection from degradation by nucleases.

mC5-MTase methylates the 5th carbon on the pyrimidine ring of cytosine in specific nucleotide motifs, creating a 5-methyl-cytosine modification [64, 65]. 5-methyl-cytosine modifications are commonly found in eukaryotic genomes and are implicated in various functions such as regulating gene expression, transposon silencing, genomic imprinting, and development [59]. This modification is also found in some virus genomes including *Phycodnaviridae* family members and is thought to provide the viral genome protection from nucleases [53, 59, 63]. The mC5-MTase encoded by CpV-BQ1 is likely required for methylation of its own genome. However, it also could be involved in manipulating its host’s cellular processes, such as downregulating the expression of host genes. To explore the CpV-BQ1 genome methylation landscape single-molecule real-time sequencing could be used to identify both N^6^-methyl-adenine and 5-methyl-cytosine modifications and gain deeper insight into the protection and gene regulation mechanisms provided by these MTases [59, 66].

FkbM methyltransferase is a S-adenosyl-L-methionine (SAM) transferase dependent methyltransferase enzyme with O-methylation activity [67]. FkbM was originally isolated from bacteria and has been shown to perform post-modification of the macrocyclic polyketides FK506, FK520, and the antibiotic rapamycin [68]. FkbM has also been found in *Phycodnaviridae* genomes, however, its substrate interactions and function is unknown [53, 54, 69]. The final methyltransferase encoded by CpV-BQ1 could not be assigned a specific function and is designated a general SAM-dependent methyltransferase [70]. SAM-MTases are a broad group of methyltransferases found in all domains of life which serve many different biological functions, thus, the functional role of this CpV-BQ1 encoded enzyme is unknown [71].

### Sugar manipulation

Three glycosyltransferase proteins were identified in the CpV-BQ1 genome. Glycosyltransferases attach sugar moieties to proteins, which can constitute important post-translational modifications. Glycosidic bonds are catalyzed using sugar donors which contain either a nucleoside phosphate or a lipid phosphate leaving group [72, 73]. Glycosyltransferases are classified into hierarchical groups based on families, clans, and fold structures [74]. A total of 137 glycosyltransferase (GT) families have been classified to date, listed on the carbohydrate-active enzymes (CAZy) database (http://www.cazy.org/*)* [75]. Of the three identified GTs in the CpV-BQ1 genome, two were classified as members of specific families, while the third could not be classified as a specific GT family member.

The GT encoded at locus BQ1_30 belongs to the family GT2. The GT2 family is one of the largest GT groups and contains members with a wide variety of catalytic activities. These enzymatic functions include, but are not limited to, cellulose synthase, chitin synthase, hyaluronan synthase, and β-glucosyltransferase [74]. GT2 family members contain a GT-A fold defined by two closely positioned β/α/β Rossmann domains, and use an inverting mechanism during catalysis of the donor substrate [73].

The GT encoded at locus BQ1_106 belongs to the family GT17, a small GT group in which all family members exhibit β-1,4-N-Acetylglucosaminyltransferase (GnTIII) activity and use an inverting mechanism during catalysis [74]. In vertebrates, GnTIII catalyzes the formation of bisecting N-acetylglucosamine (GlcNAc) residues on *N*-glycans within the Golgi apparatus. This modification inhibits the action of branching enzymes, preventing the formation of highly branched *N*-glycan structures [76, 77]. In viruses, glycans are commonly *N*-linked to a glycoprotein asparagine residue via GlcNAc [78]. These modifications are prevalent on glycoproteins in virion envelopes and play important roles in viral infection stages, including progeny formation and cellular infection [78].

Moreover, some NCLDV virions have glycans attached to the surface of major capsid proteins via the activity of GTs [79]. Thus, possible functions of the three GTs encoded by CpV-BQ1 may be the synthesis of *N*-glycans on major capsid proteins or glycoproteins in the virion envelope, however, these possibilities have yet to be explored experimentally.

### Protein and lipid binding, synthesis, and modifications

The CpV-BQ1 genome encodes two proteins that likely participate in protein synthesis, the translation initiation factor 4E (eTIF4E) and DNAJ. The eTIF4E facilitates binding of the host’s ribosome to the 5’ prime cap of mRNA which initiates protein translation [80]. DNAJ, originally identified in prokaryotes, functions as a cochaperone protein to Hsp70 and aids in protein folding [81]. However, studies of DNAJ homologs in viruses show this protein has diverse roles and can also be involved in genome replication, transcriptional activation, virion assembly, and cellular transformation [81]. Thus, further study is required to determine the exact functional nature of the DNAJ protein encoded by CpV-BQ1. The CpV-BQ1 genome also encodes three tRNAs, including tRNA-Leu, tRNA-Arg, and tRNA-Ile, which are required for incorporation of these three amino acids into nascent peptide chains [82].

Protein modification enzymes encoded by CpV-BQ1 indicate the host ubiquitination pathway is utilized by this virus to modulate proteins and perhaps subvert host defense mechanisms. Ubiquitination of proteins can either modulate their activity or signal their degradation [40]. Four E3 ubiquitin ligase proteins are encoded by the CpV-BQ1 genome. Interestingly, one of the E3-ligases encoded by the genome was denoted as a N1r/p28-like protein. The N1r/p28 protein was the first E3-ligase discovered in poxviruses, it is recruited to virus factories within the cell and is an important virulence factor [40, 83]. Thus, the CpV-BQ1 encoded N1R/p28 protein may be involved in the ubiquitination of important proteins within virus factories. Other proteins which may be involved in protein modification include six F-box domain containing proteins, which are thought to be involved in the ubiquitin-ligase complex [84], as well as, two proteins containing a metallopeptidase (WLM) domain, which are also thought to be associated with ubiquitin-signaling pathways [85].

Lastly, the patatin-like phospholipase protein encoded by CpV-BQ1 has also been found in many other NCLDVs however its function is not well understood [86]. In plants, patatin phospholipase catalyzes the cleavage of fatty lipids from membranes, while in bacteria, this protein has been implicated in the pathogen-host interaction [87, 88].

### DNA packaging and genome completeness

The CpV-BQ1 genome encodes the viral A32 protein found in all NCLDVs which is essential for the packaging of viral DNA into virions [86, 89]. Silencing of the viral A32 protein results in virion structures devoid of viral DNA [90]. A32 is thought to form a hexameric ring on the membrane surface of immature virions and pumps complete viral DNA into the virion [39].

**Fig. 8:**
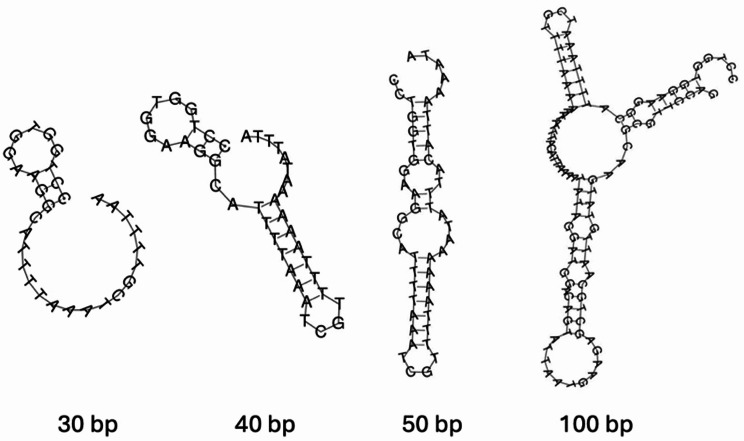
Potential hairpin structures of CpV-BQ1 genome termini. Possible secondary DNA hairpin structures formed by the 30, 40, 50, and 100 bp ends of the CpV-BQ1 genome modeled using the DNA secondary structure tool by VectorBuilder [79]

In addition to the A32 protein, packaging of many NCLDV genomes, including Poxviridae [91], ASFV [92], phycodnaviruses [30, 93, 94], and mimiviruses [8, 95] requires the presence of inverted terminal repeats on the distal ends of their genomes [96]. These inverted repeats contain, in order, genes, tandem DNA repeats, and mismatched hairpin ends which interact with packaging enzymes ensuring complete genomes are incorporated into virions [39]. The CpV-BQ1 genome has inverted repeats 3928 bp long at its distal ends which contain identifiable genes and tandem repeats. The possibility of hairpin sequences in the first 30, 40, 50, and 100 bp of the genome was investigated using the DNA secondary structure prediction tool by vector builder [97] (Fig. 8). Of the four analyzed sequences, the 50 bp sequence provides the most promising mismatched hairpin structure, as it is similar to the stem-loop organization of the vaccinia virus hairpin motifs [91].

The presence and structure of mismatched hairpin ends cannot be verified by genome assembly alone. However, based on the presence of terminal distal repeats and their well-defined role in other NCLDVs [30, 39, 95, 96] as well as preliminary secondary structural analysis, it is likely that the CpV-BQ1 genome forms hairpin structures. Most importantly, since distal inverted repeats are a signature of many NCLDV genome ends, the presence of these inverted repeats on the distal ends of the CpV-BQ1 genome provide strong evidence that the CpV-BQ1 genome has been sequenced completely.

### Virion capsid and associated structural proteins

Four putative viral capsid proteins were identified in the CpV-BQ1 genome. InterPro analysis of putative capsid proteins at locus BQ1_139, BQ1_140, and BQ1_141 identified an adenovirus hexon domain in these three proteins. The adenovirus hexon domain contains a jelly-roll fold which is a signature feature of NCLDV capsid proteins [98, 99]. Furthermore, these three proteins and BQ1_142 had a high degree of structurally similarity to other NCLDV capsid structures when analyzed with the homology-based protein fold recognition tool Phyre2 [100]. Lastly, these proteins are encoded sequentially on the same strand. This proximity would permit the synthesis of all four genes in tandem, making their synthesis highly efficient. NCLDV capsid proteins have a high degree of sequence variation; a protein Blast analysis of these four proteins did not detect any sequence homology with other proteins [46]. Thus, if these putative capsid proteins are indeed structural components of the virion capsid, this would expand our knowledge of NCLDV capsid sequences and aid in the identification of other capsids. Additionally, two minor capsid proteins were identified in the CpV-BQ1 genome. The minor capsid P9 transmembrane helices containing protein has domains similar to the inner capsid P9 structure of the NCLDV PBCV-1 [99]. The second minor protein is a tape measure protein, which likely spans from one virion icosahedral vertex to another and is thought to play a critical role in mediating the capsid size and maintaining the orientation of capsomers to one another during assembly [101].

The CpV-BQ1 genome also encodes two tail fiber proteins, one of which contains a fibronectin III binding domain. Tail fiber proteins are found in bacteriophages and facilitate the injection of viral DNA into cells. However, they have also been found in some NCLDVs including in chloroviruses and other phycodnaviruses [54, 102]. In PBCV-1, tail fiber proteins form a spike on a vertice of the icosahedral capsid structure which interacts with the host cell to facilitate virion entry [102, 103]. Similarly, the CpV-BQ1 genome tail fiber proteins may form a spike on the capsid to facilitate cellular entry.

An important post-translational NCLDV capsid protein modification for assembly and stabilization in ER membrane encompassed virion factories is the formation of disulfide bonds between conserved cysteine residues [40, 104]. These bonds are catalyzed by a redox reaction requiring two enzymes, a thioredoxin domain containing protein and an ERV sulfhydryl oxidase [56, 104]. The CpV-BQ1 genome encodes an ERV/ALR sulfhydryl oxidase and two thioredoxin domain containing proteins, a thioredoxin and a glutaredoxin-like protein. DeepLoc subcellular predictions identified ER signaling domains on the ERV/ALR sulfhydryl oxidase and glutaredoxin-like protein. Together, these two proteins likely complete the redox reaction necessary for assembly and stabilization of capsid structures. DeepLoc predicted the thioredoxin protein is within the cytoplasmic compartment; however, this does not definitively exclude it from involvement in the capsid redox cascade reaction. Lastly, the CpV-BQ1 genome encodes an Ac78 gene which is similar to the baculovirus protein Ac78 [105]. This protein is important for budded virion production, embedding of virions into occlusion bodies, and primary cellular infection. It has been shown to play an important role in virion localization however is not essential for virion assembly or structure [105].

### Comparison of CpV-BQ1 and CpV-BQ2 genomes

With the publication of this work we now have the full genome sequence of two viruses, CpV-BQ1 (this study) and CpV-BQ2 [27], which can infect *C. parva*. CpV-BQ1 and CpV-BQ2 are taxonomically assigned to the NCLDV families *Phycodnaviridae* and *Mesomimiviridae* respectively. One striking difference between CpV-BQ1 and CpV-BQ2 is their genome size. CpV-BQ1 has a 165,454 bp genome with 32.32% GC content that encodes 193 ORFs, whereas CpV-BQ2 has a 437,255 bp genome with 25% GC content that encodes for 503 ORFs [27]. This difference in both size and coding potential indicates that although they share the same host, they likely use remarkably different infection and replication strategies. For example, CpV-BQ2 encodes at least eight restriction-modification (R-M) systems and 13 methyltransferases [27], whereas CpV-BQ1 does not encode any R-M systems and only encodes 4 methyltransferases. This indicates these viruses use very different methods to protect their own DNA and/or degrade host DNA which can alter cellular processes such as metabolism, transcription, and translation [59, 60]. Furthermore, the presence of an RNA polymerase (RNA pol) II gene in CpV-BQ2 and its absence in CpV-BQ1 indicate these viruses use very different infection and replication strategies. RNA pol encoding viruses can transcribe their own DNA, NCLDVs that encode their own RNA pol typically carry out transcription, genome replication, and virion packaging all within the host’s cytoplasm [42]. On the other hand, NCLDVs that do not encode an RNA pol require a nuclear infection step to hijack their host’s transcriptional machinery which is followed by a cytoplasmic infection wherein virion packaging occurs [43]. Thus, CpV-BQ1 likely utilizes a two-step infection/replication strategy that includes both cytoplasmic and nuclear infection which requires coordination across subcellular compartments, whereas CpV-BQ2 likely infects *C. parva* through a one-step cytoplasmic approach.

## Conclusions

In this study we have sequenced and annotated the complete linear 165,454 bp genome of Chrysochromulina parva virus BQ1 (CpV-BQ1). CpV-BQ1 was originally isolated from a lake in Ontario and is a lytic agent of the haptophyte alga *C. parva.* Taxonomic analysis of *polB*, A32 ATPase, and VLTF-3 protein sequences were used to assign the *Phycodnaviridae* family classification to CpV-BQ1. The genome contains 193 genes, of which 92 could be assigned a known function. CpV-BQ1 has hallmark genes found in many NCLDVs necessary for genome replication, virion production, and transcription [3]. Like other phycodnaviruses, CpV-BQ1 most likely has a two-step cellular infection life-cycle, first entering the nucleus for transcription as evidenced by the lack of an RNA polymerase gene, then virion assembly ensues within the cytoplasm [43, 51]. Previously, another *C. parva* lytic agent Chrysochromulina parva virus BQ2 (CpV-BQ2) was isolated from the same water sample as CpV-BQ1 [25], its genome was sequenced and was taxonomically assigned to the *Mesomimiviridae* family [27]. Thus far, co-infection of a eukaryotic algae with viruses from both *Phycodnaviridae* and *Mesomimivirdae* families has only been observed in the species *Phaeocystis globosa* [38, 106]. However, only the complete genome sequence of one *P. globosa* infecting virus, the group II PgV-16T mimivirus, has been sequenced and made available [107]. It is postulated that co-infections of eukaryotic algae with viruses belonging to different NCLDV families is common, however, due to a lack of data this hypothesis cannot be currently supported [106]. Indeed, only ~ 60 eukaryotic algal viruses have been isolated in culture [106], while thousands of eukaryotic algal species have been isolated and are available in culture collections around the world. This disparity emphasizes the lack of research and knowledge surrounding eukaryotic algal virus diversity, taxonomy, life cycle, and environmental impact. With the work reported here and by Stough et al. 2019 [27], we have for the first time established an algal-virus system with complete genome sequences for both *Phycodnaviridae* (this study) and *Mesomimiviridae* [27] viruses. This system can be used to study the biological, ecological, and environmental consequences of the coding potential of these viruses which replicate in the same host. For example, the presence of RM systems encoded by CpV-BQ2 and their absence in CpV-BQ1 may be relevant to inter-viral competition and suggest that BQ2 may restrict the replication of BQ1, or the PLVs which presumably parasitize one of these viruses. With the genomic information in hand, detailed transcriptional studies can be conducted to further understand the complicated dynamics and relationships between *C. parva* and its viral parasites. In turn, this knowledge will illuminate the potential complexities of algal virus-host interactions and is especially important considering the critical importance of algae in the biosphere and human affairs.

## Materials and methods

### Host and virus cultivation

Viruses infecting *Chrysochromulina parva* strain CCMP 291 (National Center for Marine Algae and Microbiota, East Boothbay, Maine, USA) were originally isolated from an embayment of Lake Ontario, Canada, in 2011 [25] and have been regularly propagated in the laboratory since then. The CpV-BQ1 virus was purified from cultures of mixed *C. parva* viruses, as described in Stough et al. (2019), via an end-point dilution approach. Briefly, serial 10-fold dilutions of *C. parva* lysates were inoculated into 96-well microtiter plates with mid-log phase *C. parva* cells at a concentration of approximately 6.0 × 10^5^ cells/mL as determined using a hemocytometer and light microscope. Medium from individual wells which lysed at the highest dilution level (i.e., lowest concentration) of viruses were transferred in into 50 mL cultures of mid-log *C. parva* and the resulting lysates were filtered through sterile 0.22 μm pore-size PVDF Durapore^®^ membrane filters (EMD Millipore, GVWP00010). This process was repeated 3 times to ensure that only a single type of CpV was propagated, and the presence of CpV-BQ1 was confirmed throughout this purification process using the qPCR method described in Mirza et al. (2015).

### Transmission electron microscopy

Following ultracentrifugation steps noted above, 10 µL of the concentrated CpV-BQ1 sample was applied to a formvar and carbon-coated copper grid (FCF400-Cu-UB, Electron Microscopy Sciences) which was glow discharged for 10 min immediately before sample application. Following sample application, the grid was washed three times on droplets of double distilled H_2_O and was placed on a droplet of 2% Uranyl Acetate for 30 s. At each step, excess stain was wicked away. Stained grids were visualized using a Talos L120C transmission electron microscope (Thermo Fisher Scientific) at the Microscopy Imaging Laboratory, Temerty Faculty of Medicine, University of Toronto, Canada. ImageJ was used for subsequent image analysis.

### Nucleic acid extraction

Following purification of CpV-BQ1, genomic material was prepared for sequencing by extracting nucleic acids from a 600 mL lysate of a *C. parva* culture infected with CpV-BQ1. This lysate was filtered through a 0.22 μm pore-size Steritop^®^ disposable bottle top filters (MilliporeSigma, S2GPT10RE) and was concentrated via ultracentrifugation at 31,000 rpm for 1 h at 20 °C in a SW32Ti rotor (Beckman Coulter Life Sciences). The supernatant was decanted and pelleted material was resuspended in 10 mM Tris-Cl, pH 8.5. Nucleic acids were extracted from the concentrated viral lysate using a Maxwell^®^ RSC Viral Total Nucleic Acid extraction kit (Promega, AS1330) following the manufacturer’s protocol for 300 µL of sample input. The DNA concentration measured using a Qubit fluorometer with a dsDNA HS kit (Thermo Fisher Scientific, Q32851) was 26.2 ng/µL.

### Illumina and nanopore library preparation and sequencing

The DNA was sent to SeqCenter (Pittsburgh, USA) for paired-end whole genome shotgun sequencing. Sample libraries were prepared using the Illumina DNA Prep kit and IDT 10 bp UDI indices, and were sequenced on an Illumina NovaSeq 6000, producing 2 × 151 bp reads. Demultiplexing, quality control, and adapter trimming was performed with BCL-Convert v4.0.3, generating 14,757,009 read pairs.

The DNA was also prepared for Nanopore sequencing at the University of Waterloo. Extracted DNA was diluted to a concentration of 5 ng/µL and prepared with the Nanopore Rapid PCR barcoding 24 V14 kit (Nanopore, SQK-RPB114.24). The prepared DNA library was sequenced on a MinION Mk1B device using an R10.4.1 flow cell (Nanopore, FLO-MIN114).

### Read processing and genome assembly

Nanopore sequencing produced a total of 2,325,374 raw reads. First, Filtlong was used to remove reads less than 1000 bp long and 10% of the worst quality reads, resulting in 1,879,244 reads [108]. Kraken2 v2.0.7-beta was then used to filter out classified reads, removing contaminating host, bacterial, and human reads. The standard Kraken2 database v9/26/2022 was used with a 0.001 confidence filter and the unclassified-out flag to generate a separate file of unclassified reads, from which 1,048,312 reads were obtained [109].

Filtered long reads and Illumina reads were then used for assembly with the TryCycler pipeline v0.5.4 [31]. Briefly, Filtlong was used to keep the best 80% of filtered nanopore reads. This final read filtering step yielded 796,152 reads with an N50 of 6,479 bp and a mean length of 6,352 bp [108]. Long reads were then subsampled to create 24 read subsets with approximately 8,645 reads per subset. An estimated genome size of 400,000 bp was used to subset reads, which was based on the size of the CpV-BQ2 virus genome (Accession MH918795). Each read subset was assembled with either Flye v2.9.2-b1786, Miniasm & Minipolish v0.1.2, Raven v1.8.1, or Canu v2.2 [33–36]. Of the 24 read subsets, 6 were assembled into contigs by each respective tool [33–36]. From these 24 genome assemblies, 26 contigs were produced.

Assembled contigs were clustered to determine the MASH distance between assemblies. The MASH distance is based on the Jaccard index and provides a measure of similarity and diversity between each sample. Using MASH distances, a phylogenetic tree was generated during the TryCycler clustering step (Fig. 2). Of the 26 assembled contigs, 23 clustered closely together. These assembled contigs were reconciled which ensures contigs are on the same strand and sufficiently similar for downstream assembly. To ensure contigs were sufficiently similar, the TryCycler default thresholds were used, including a 98% sequence identity score and minimum 1-kbp identity score of 25%. Based on these thresholds, 7 of the 23 contigs were discarded. A dotplot was generated to visualize the topology of the remaining 16 contigs (Fig. 3).

A multiple sequence alignment was generated from the remaining 16 clustered and reconciled contigs with the MUSCLE algorithm [110]. Long reads were partitioned to each assembly to determine the single best alignment for each read. Then, a linear consensus sequence was generated based on the best read alignments. Long-read correction with the Nanopore tool Medaka v1.11.1 was used to polish the consensus sequence [111, 112]. Short Illumina read pairs were quality filtered using Fastp v0.23.4. Then, two iterations of polypolish v0.5.0 were used to correct genome errors using the filtered Illumina reads and generate the final genome assembly [113, 114]. Alignment of Illumina reads to the assembled genome produced a mean read depth of 21,688.3x, corrected 54 positions in the genome, and identified a 104 bp region at the C-terminus that had 0% coverage. This 104 bp region was removed from the final assembly, and likely arose from erroneous read extension. The final assembly resulted in a genome length of 165,454 bp.

### Primer design, PCR amplification, and analysis of random genomic regions

The CpV-BQ1 assembly was verified by PCR amplification and sequencing of five regions, approximately 9,000 bp in length, across the genome (Fig. 4). To design primers, the CpV-BQ1 genome was uploaded to PrimalScheme and an amplicon size of 9,000 bp was selected [115]. PrimalScheme generated 19 potential primer pairs that could be used across the entire genome, of which five were selected to perform random spot checks across the genome (Supplementary Table 1). An additional four primers were designed to sequence across the genome ends in order to investigate the genome topology (Fig. 4). PCR amplification was performed with 2x GB-AMP PaCeR HP Master Mix (GeneBio Systems, PCR-002-01), 0.4 µM of forward and reverse primers, and ~ 1.1 ng of genomic DNA. Thermocycling conditions were: initial denaturation at 95 °C for 30 s, 35 cycles of 95 °C for 15 s, 68 °C for 15 s, and 72 °C for 10 min, then a final extension at 72 °C for 6.5 min and samples were held at 12 °C. PCR products were analyzed by gel electrophoresis on a 1% agarose gel and concentration was evaluated with a Qubit 4.0 fluorometer. PCR fragments were cleaned up with AMPure XP beads (Beckman-Coulter, A63881) to remove primers and small DNA fragments. Recovered products were prepared using the Nanopore Rapid Barcoding kit V14 (Nanopore, SQK-RBK114.24). The prepared library was loaded onto a MinION R10.4.1 flowcell (Nanopore, FLO-MIN114) and sequenced on a Nanopore MinION. Reads were analyzed using the Trycycler pipeline as previously described with a few exceptions. Reads were filtered once using filtlong, twelve read subsets were generated using a genome size of 9,000 bp, and only one round of polishing was performed with polypolish. Assembled contigs (Supplementary Table 2) were aligned to the CpV-BQ1 genome using the MUSCLE algorithm and visualized with Seaview [110, 116].

### Open reading frame (ORF) prediction

Open reading frames (ORFs) were predicted using a combination of four prediction tools: GeneMarkS, Glimmer3, Prodigal, and FragGeneScan [117–120]. Any ORF predicted by two or more tools was considered a putative gene encoding ORF [121–123]. When differing gene start sites were identified, the positions identified with GeneMarkS were used first, followed by Prodigal, then FragGeneScan [122]. A total of 193 ORFs were predicted, of which GeneMarkS, Prodigal, FragGeneScan, and Glimmer3 predicted 98.45%, 95.85%, 88.08%, and 97.4% of these ORFs, respectively (Supplementary Table 3).

### Gene annotation

ORFs were assessed for functional predictions using multiple approaches, including database searches, InterPro analysis, and HMM classification. A BLASTp search against the non-redundant protein sequence database was performed twice, once including all organisms and a second search against only viruses [124]. Additionally, a search was performed against the IMG virus protein BLAST database [125]. InterPro was used to predict protein functionality using protein domain and family predictions [126]. InterPro has multiple tools integrated into its online platform including Panther, NCBIfam, CDD, Cath-Gene3D, SUPERFAMILY, ProSiteProfiles, ProSitePatterns, PRINTS, SMART, Pfam, HHMAP, and FunFam to classify protein families, domains, superfamilies, and amino acid sites. InterPro also identifies signal peptides, transmembrane helices, coils, and disordered regions using TMHMM, Coils, MobliteDB, and Phobius. HMM profile analysis against the PhROGs, efam-xc, RVDB, and VOGDB were performed to classify ORFs [127–130]. MMSeqs was used to perform HMM analysis against the PhROGs database while HMMer3 was used to search all other HMM databases [131, 132]. tRNAscan-SE was used to predict tRNA sequences in the assembled CpV-BQ1 genome [82].

### Taxonomic classification

Three genes common to all NCLDVs were used to taxonomically classify the CpV-BQ1 genome, DNA polymerase type-B, viral A32-like packaging ATPase, and viral late transcription factor 3 (VLTF-3). Protein sequences and accessions for each gene from various NCLDVs were downloaded from NCBI (Supplementary Table 4). Multiple sequence alignments (MSAs) were generated using the MUSCLE algorithms for each protein using Seaview v5.0.4 [110, 116]. MSAs were used to generate phylogenetic trees in Seaview using the phylogenetic maximum likelihood method with the LG model, aLRT (SH-like) branch support, NNI tree searching operation, and a BioNJ starting tree [116, 133].

## Electronic supplementary material

Below is the link to the electronic supplementary material.

Supplementary Material 1

Supplementary Material 2

Supplementary Material 3

Supplementary Material 4

## Data Availability

The CpV-BQ1 genome sequence is available on GenBank under accession PQ783904. Raw reads are available on the SRA database BioProject accession number PRJNA1199504. BioSample accessions for raw reads are as follows: Illumina whole genome sequences, SAMN45876274; Nanopore whole genome sequences, SAMN45876275; CpV-BQ1 PCR amplified region 1, SAMN45876276; CpV-BQ1 PCR amplified region 2, SAMN45876277; CpV-BQ1 PCR amplified region 3, SAMN45876278; CpV-BQ1 PCR amplified region 4, SAMN45876279; CpV-BQ1 PCR amplified region 5, SAMN45876280.

## References

[1] Schulz F, Roux S, Paez-Espino D, Jungbluth S, Walsh DA, Denef VJ, et al. Giant virus diversity and host interactions through global metagenomics. Nature. 2020;578(7795):432–6. 31968354 10.1038/s41586-020-1957-x PMC7162819

[2] Aylward FO, Abrahão JS, Brussaard CPD, Fischer MG, Moniruzzaman M, Ogata H, et al. Taxonomic update for giant viruses in the order imitervirales (phylum Nucleocytoviricota). Arch Virol. 2023;168(11):283. 37904060 10.1007/s00705-023-05906-3 PMC11230039

[3] Mönttinen HAM, Bicep C, Williams TA, Hirt RP. The genomes of nucleocytoplasmic large DNA viruses: viral evolution writ large. Microb Genomics. 2021;7(9). 10.1099/mgen.0.000649 PMC8715426 34542398

[4] Homola M, Büttner CR, Füzik T, Křepelka P, Holbová R, Nováček J et al. Structure and replication cycle of a virus infecting climate-­modulating Alga emiliania huxleyi. Sci Adv. 2024;10(15). 10.1126/sciadv.adk1954 PMC11006232 38598627

[5] Koonin EV, Yutin N. Origin and evolution of eukaryotic large Nucleo-Cytoplasmic DNA viruses. Intervirology. 2010;53(5):284–92. 20551680 10.1159/000312913 PMC2895762

[6] Yoosuf N, Yutin N, Colson P, Shabalina SA, Pagnier I, Robert C, et al. Related giant viruses in distant locations and different habitats: Acanthamoeba polyphaga moumouvirus represents a third lineage of the mimiviridae that is close to the megavirus lineage. Genome Biol Evol. 2012;4(12):1324–30. 23221609 10.1093/gbe/evs109 PMC3542560

[7] Moniruzzaman M, Martinez-Gutierrez CA, Weinheimer AR, Aylward FO. Dynamic genome evolution and complex virocell metabolism of globally-distributed giant viruses. Nat Commun. 2020;11(1). 10.1038/s41467-020-15507-2 PMC7136201 32249765

[8] Raoult D, Audic S, Robert C, Abergel C, Renesto P, Ogata H, et al. The 1.2-Megabase genome sequence of mimivirus. Science. 2004;306(5700):1344–50. 15486256 10.1126/science.1101485

[9] Scola BL, Audic S, Robert C, Jungang L, De Lamballerie X, Drancourt M, et al. A giant virus in amoebae. Science. 2003;299(5615):2033–2033. 12663918 10.1126/science.1081867

[10] Colson P, Yutin N, Shabalina SA, Robert C, Fournous G, La Scola B, et al. Viruses with more than 1,000 genes: mamavirus, a new Acanthamoeba polyphagamimivirus strain, and reannotation of mimivirus genes. Genome Biol Evol. 2011;3:737–42. 21705471 10.1093/gbe/evr048 PMC3163472

[11] Hingamp P, Grimsley N, Acinas SG, Clerissi C, Subirana L, Poulain J, et al. Exploring nucleo-cytoplasmic large DNA viruses in Tara oceans microbial metagenomes. ISME J. 2013;7(9):1678–95. 23575371 10.1038/ismej.2013.59 PMC3749498

[12] Kim KE, Joo HM, Lee TK, Kim HJ, Kim YJ, Kim BK et al. Covariance of marine nucleocytoplasmic large dna viruses with eukaryotic plankton communities in the sub-arctic Kongsfjorden ecosystem: a metagenomic analysis of marine microbial ecosystems. Microorganisms. 2023;11(1). 10.3390/microorganisms11010169 PMC9862967 36677461

[13] Laber CP, Hunter JE, Carvalho F, Collins JR, Hunter EJ, Schieler BM, et al. Coccolithovirus facilitation of carbon export in the North Atlantic. Nat Microbiol. 2018;3(5):537–47. 29531367 10.1038/s41564-018-0128-4

[14] Liang JL, Feng S, wei, Jia P, Lu J, li, Yi X, Gao S et al. ming,. Unraveling the habitat preferences, ecological drivers, potential hosts, and auxiliary metabolism of soil giant viruses across China. Microbiome. 2024;12(1). 10.1186/s40168-024-01851-8 PMC11265010 39039586

[15] Endo H, Blanc-Mathieu R, Li Y, Salazar G, Henry N, Labadie K, et al. Biogeography of marine giant viruses reveals their interplay with eukaryotes and ecological functions. Nat Ecol Evol. 2020;4(12):1639–49. 32895519 10.1038/s41559-020-01288-w

[16] Cottrell M, Suttle C. Wide-spread occurrence and clonal variation in viruses which cause Lysis of a cosmopolitan, eukaryotic marine phytoplankter Micromonas pusilla. Mar Ecol Prog Ser. 1991;78:1–9.

[17] Tarutani K, Nagasaki K, Itakura S, Yamaguchi M. Isolation of a virus infecting the novel shellfish-killing dinoflagellate Heterocapsa circularisquama. Aquat Microb Ecol. 2001;23:103–11.

[18] Brussaard CPD, Short SM, Frederickson CM, Suttle CA. Isolation and phylogenetic analysis of novel viruses infecting the phytoplankton Phaeocystis globosa (Prymnesiophyceae). Appl Environ Microbiol. 2004;70(6):3700–5. 15184176 10.1128/AEM.70.6.3700-3705.2004 PMC427783

[19] Wheeler GL, Sturm D, Langer G. *Gephyrocapsa huxleyi* (*Emiliania huxleyi*) as a model system for coccolithophore biology. J Phycol. 2023;59(6):1123–9. 37983837 10.1111/jpy.13404

[20] Highfield A, Evans C, Walne A, Miller PI, Schroeder DC. How many coccolithovirus genotypes does it take to terminate an emiliania huxleyi bloom? Virology. 2014;466–467:138–45. 25085627 10.1016/j.virol.2014.07.017

[21] Nissimov JI, Vandzura R, Johns CT, Natale F, Haramaty L, Bidle KD. Dynamics of transparent exopolymer particle production and aggregation during viral infection of the coccolithophore, *Emiliania huxleyi*. Environ Microbiol. 2018;20(8):2880–97. 29921002 10.1111/1462-2920.14261

[22] Nicholls KH. Chapter 13 - Haptophyte algae. Freshwater algae of North America. Academic; 2015. pp. 587–605.

[23] Parke M, Lund JWG, Manton I. Observations on the biology and fine structure of the type species of Chrysochromulina (C. parva Lackey) in the english lake district. Arch Für Mikrobiol. 1962;42(4):333–52. 10.1007/BF00409070 14483908

[24] Hovde BT, Deodato CR, Andersen RA, Starkenburg SR, Barlow SB, Cattolico RA. Chrysochromulina: genomic assessment and taxonomic diagnosis of the type species for an oleaginous algal clade. Algal Res. 2019;37:307–19.

[25] Mirza SF, Staniewski MA, Short CM, Long AM, Chaban YV, Short SM. Isolation and characterization of a virus infecting the freshwater algae Chrysochromulina parva. Virology. 2015;486:105–15. 26432023 10.1016/j.virol.2015.09.005

[26] Rozon RM, Short SM. Complex seasonality observed amongst diverse phytoplankton viruses in the Bay of Quinte, an embayment of lake Ontario. Freshw Biol. 2013;58(12):2648–63.

[27] Stough JMA, Yutin N, Chaban YV, Moniruzzaman M, Gann ER, Pound HL et al. Genome and environmental activity of a Chrysochromulina parva virus and its virophages. Front Microbiol. 2019;10. 10.3389/fmicb.2019.00703 PMC6459981 31024489

[28] Chaudhari HV, Inamdar MM, Kondabagil K. Scaling relation between genome length and particle size of viruses provides insights into viral life history. iScience. 2021;24(5):102452. 34113814 10.1016/j.isci.2021.102452 PMC8169800

[29] Milrot E, Mutsafi Y, Fridmann-Sirkis Y, Shimoni E, Rechav K, Gurnon JR, et al. Virus-host interactions: insights from the replication cycle of the large *Paramecium bursaria chlorella virus*: replication factories of PBCV-1. Cell Microbiol. 2016;18(1):3–16. 26248343 10.1111/cmi.12486

[30] Rey Redondo E, Leung SKK, Yung CCM. Genomic and biogeographic characterisation of the novel prasinovirus *Mantoniella Tinhauana* virus 1. Environ Microbiol Rep. 2024;16(5):e70020. 39392286 10.1111/1758-2229.70020 PMC11467894

[31] Wick RR, Judd LM, Cerdeira LT, Hawkey J, Méric G, Vezina B et al. Trycycler: consensus long-read assemblies for bacterial genomes. Genome Biol. 2021;22. 10.1186/s13059-021-02483-z PMC8442456 34521459

[32] Sohn Jil, Nam JW. The present and future of de Novo whole-genome assembly. Brief Bioinform. 2016;19(1):23–40. 10.1093/bib/bbw096 27742661

[33] Koren S, Walenz BP, Berlin K, Miller JR, Bergman NH, Phillippy AM. Canu: scalable and accurate long-read assembly via adaptive *k* -mer weighting and repeat separation. Genome Res. 2017;27(5):722–36. 28298431 10.1101/gr.215087.116 PMC5411767

[34] Kolmogorov M, Yuan J, Lin Y, Pevzner PA. Assembly of long, error-prone reads using repeat graphs. Nat Biotechnol. 2019;37(5):540–6. 30936562 10.1038/s41587-019-0072-8

[35] Wick RR, Holt KE. Benchmarking of long-read assemblers for prokaryote whole genome sequencing. F1000Research. 2021;8. 10.12688/f1000research.21782.1 PMC6966772 31984131

[36] Vaser R, Šikić M. Time- and memory-efficient genome assembly with Raven. Nat Comput Sci. 2021;1(5):332–6. 38217213 10.1038/s43588-021-00073-4

[37] Suttle CA, Chan AM. Viruses infecting the marine prymnesiophyte Chrysochromulina spp.: isolation, preliminary characterization and natural abundance. Mar Ecol Prog Ser. 1995;118:275–82.

[38] Baudoux AC, Brussaard CPD. Characterization of different viruses infecting the marine harmful algal bloom species Phaeocystis globosa. Virology. 2005;341(1):80–90. 16081120 10.1016/j.virol.2005.07.002

[39] Greseth MD, Traktman P. The life cycle of the vaccinia virus genome. Annu Rev Virol. 2022;9(1):239–59. 35584888 10.1146/annurev-virology-091919-104752

[40] Iyer LM, Balaji S, Koonin EV, Aravind L. Evolutionary genomics of nucleo-cytoplasmic large DNA viruses. Virus Res. 2006;117(1):156–84. 16494962 10.1016/j.virusres.2006.01.009

[41] Ødum MT, Teufel F, Thumuluri V, Almagro Armenteros JJ, Johansen AR, Winther O, et al. DeepLoc 2.1: multi-label membrane protein type prediction using protein Language models. Nucleic Acids Res. 2024;52(W1):W215–20. 38587188 10.1093/nar/gkae237 PMC11223819

[42] Moniruzzaman M, Erazo Garcia MP, Farzad R, Ha AD, Jivaji A, Karki S et al. Virologs, viral mimicry, and virocell metabolism: the expanding scale of cellular functions encoded in the complex genomes of giant viruses. FEMS Microbiol Rev. 2023;47(5). 10.1093/femsre/fuad053 PMC10583209 37740576

[43] Moreau H, Piganeau G, Desdevises Y, Cooke R, Derelle E, Grimsley N. Marine prasinovirus genomes show low evolutionary divergence and acquisition of protein metabolism genes by horizontal gene transfer. J Virol. 2010;84(24):12555–63. 20861243 10.1128/JVI.01123-10 PMC3004350

[44] O’Brien MJ, Ansari A. Critical involvement of TFIIB in viral pathogenesis. Front Mol Biosci. 2021;8. 10.3389/fmolb.2021.669044 PMC8119876 33996913

[45] Hubbs AE, Wright CF. The A2L intermediate gene product is required for in vitro transcription from a vaccinia virus late promoter. J Virol. 1996;70(1):327–31. 8523544 10.1128/jvi.70.1.327-331.1996 PMC189821

[46] Iyer LM, Aravind L, Koonin EV. Common origin of four diverse families of large eukaryotic DNA viruses. J Virol. 2001;75(23):11720–34. 11689653 10.1128/JVI.75.23.11720-11734.2001 PMC114758

[47] Keck JG, Overexpression. Purification, and late transcription factor activity of the 17-Kilodalton protein encoded by the vaccinia virus all gene. J Virol. 1993;67(10):5740–8. 10.1128/jvi.67.10.5740-5748.1993 PMC237991 8371339

[48] Kettenberger H, Armache KJ, Cramer P. Architecture of the RNA polymerase II-TFIIS complex and implications for mRNA cleavage. Cell. 2003;114(3):347–57. 12914699 10.1016/s0092-8674(03)00598-1

[49] Benarroch D, Qiu ZR, Schwer B, Shuman S. Characterization of a mimivirus RNA cap guanine-N2 methyltransferase. RNA. 2009;15(4):666–74. 19218551 10.1261/rna.1462109 PMC2661837

[50] Zhang Y, Calin-Jageman I, Gurnon JR, Choi TJ, Adams B, Nicholson AW, et al. Characterization of a chlorella virus PBCV-1 encoded ribonuclease III. Virology. 2003;317(1):73–83. 14675626 10.1016/j.virol.2003.08.044

[51] Van Etten JL, Agarkova IV, Dunigan DD. Chloroviruses Viruses. 2019;12(1):20. 10.3390/v12010020 PMC7019647 31878033

[52] De Souza RF, Iyer LM, Aravind L. Diversity and evolution of chromatin proteins encoded by DNA viruses. Biochim Biophys Acta BBA - Gene Regul Mech. 2010;1799(3–4):302–18. 10.1016/j.bbagrm.2009.10.006 PMC3243496 19878744

[53] Weynberg K, Allen M, Wilson W. Marine prasinoviruses and their tiny plankton hosts: A review. Viruses. 2017;9(3):43. 28294997 10.3390/v9030043 PMC5371798

[54] Weynberg KD, Allen MJ, Gilg IC, Scanlan DJ, Wilson WH. Genome sequence of Ostreococcus Tauri virus OtV-2 throws light on the role of Picoeukaryote niche separation in the ocean. J Virol. 2011;85(9):4520–9. 21289127 10.1128/JVI.02131-10 PMC3126241

[55] Rajagopal I, Ahn BY, Moss B, Mathews CK. Roles of vaccinia virus ribonucleotide reductase and glutaredoxin in DNA precursor biosynthesis. J Biol Chem. 1995;270(46):27415–8. 7499196 10.1074/jbc.270.46.27415

[56] White CL, Senkevich TG, Moss B. Vaccinia virus G4L glutaredoxin is an essential intermediate of a cytoplasmic disulfide bond pathway required for virion assembly. J Virol. 2002;76(2):467–72. 11752136 10.1128/JVI.76.2.467-472.2002 PMC136847

[57] Bessman MJ, Frick DN, O’Handley SF. The mutt proteins or nudix hydrolases, a family of versatile, widely distributed, housecleaning enzymes. J Biol Chem. 1996;271(41):25059–62. 8810257 10.1074/jbc.271.41.25059

[58] Kago G, Parrish S. The Mimivirus L375 Nudix enzyme hydrolyzes the 5’ mRNA cap. Yang Z, editor. PLOS ONE. 2021;16(9). 10.1371/journal.pone.0245820 PMC8478210 34582446

[59] Jeudy S, Rigou S, Alempic JM, Claverie JM, Abergel C, Legendre M. The DNA methylation landscape of giant viruses. Nat Commun. 2020;11(1). 10.1038/s41467-020-16414-2 PMC7253447 32461636

[60] Agarkova IV, Dunigan DD, Van Etten JL. Virion-Associated restriction endonucleases of chloroviruses. J Virol. 2006;80(16):8114–23. 16873267 10.1128/JVI.00486-06 PMC1563800

[61] Timinskas A, Butkus V, Janulaitis A. Sequence motifs characteristic for DNA [cytosine-N4] and DNA [adenine-N6] methyltransferases. Classification of all DNA methyltransferases. Gene. 1995;157(1–2):3–11. 7607512 10.1016/0378-1119(94)00783-o

[62] Ratel D, Ravanat JL, Berger F, Wion D. N6-methyladenine: the other methylated base of DNA. BioEssays. 2006;28(3):309–15. 16479578 10.1002/bies.20342 PMC2754416

[63] Van Etten JL, Dunigan DD. Chloroviruses: not your everyday plant virus. Trends Plant Sci. 2012;17(1):1–8. 22100667 10.1016/j.tplants.2011.10.005 PMC3259250

[64] Cheng X. Structure and function of DNA methyltransferases. Annu Rev Biophys. 1995;24:293–318. 10.1146/annurev.bb.24.060195.001453 7663118

[65] Kumar S, Cheng X, Klimasauskas S, Sha M, Posfai J, Roberts RJ, et al. The DNA (cytosine-5) methyltransferases. Nucleic Acids Res. 1994;22(1):1–10. 8127644 10.1093/nar/22.1.1 PMC307737

[66] Flusberg BA, Webster DR, Lee JH, Travers KJ, Olivares EC, Clark TA, et al. Direct detection of DNA methylation during single-molecule, real-time sequencing. Nat Methods. 2010;7(6):461–5. 20453866 10.1038/nmeth.1459 PMC2879396

[67] Ban YH, Shinde PB, Hwang Jyeon, Song MC, Kim DH, Lim SK, et al. Characterization of FK506 biosynthetic intermediates involved in Post-PKS elaboration. J Nat Prod. 2013;76(6):1091–8. 23706030 10.1021/np4001224

[68] Chen D, Zhang L, Pang B, Chen J, Xu Z, Abe I, et al. FK506 maturation involves a cytochrome P450 Protein-Catalyzed Four-Electron C-9 oxidation in parallel with a C-31 *O* -Methylation. J Bacteriol. 2013;195(9):1931–9. 23435975 10.1128/JB.00033-13 PMC3624582

[69] Weynberg KD, Allen MJ, Ashelford K, Scanlan DJ, Wilson WH. From small hosts come big viruses: the complete genome of a second *Ostreococcus Tauri* virus, OtV-1. Environ Microbiol. 2009;11(11):2821–39. 19650882 10.1111/j.1462-2920.2009.01991.x

[70] Schubert HL, Blumenthal RM, Cheng X. Many paths to methyltransfer: a chronicle of convergence. Trends Biochem Sci. 2003;28(6):329–35. 12826405 10.1016/S0968-0004(03)00090-2 PMC2758044

[71] Martin JL, McMillan FM. SAM (dependent) I AM: the S-adenosylmethionine-dependent methyltransferase fold. Curr Opin Struct Biol. 2003;13(1). 10.1016/s0959-440x(02)00391-3 12504684

[72] Campbell JA, Davies GJ, Bulone V, Henrissat B. A classification of nucleotide-diphospho-sugar glycosyltransferases based on amino acid sequence similarities. Biochem J. 1997;326(3):929–39. 9334165 10.1042/bj3260929u PMC1218753

[73] Lairson LL, Henrissat B, Davies GJ, Withers SG. Glycosyltransferases: structures, functions, and mechanisms. Annu Rev Biochem. 2008;77(1):521–55. 18518825 10.1146/annurev.biochem.76.061005.092322

[74] Coutinho PM, Deleury E, Davies GJ, Henrissat B. An evolving hierarchical family classification for glycosyltransferases. J Mol Biol. 2003;328(2):307–17. 12691742 10.1016/s0022-2836(03)00307-3

[75] Drula E, Garron ML, Dogan S, Lombard V, Henrissat B, Terrapon N. The carbohydrate-active enzyme database: functions and literature. Nucleic Acids Res. 2022;50(D1):D571–7. 34850161 10.1093/nar/gkab1045 PMC8728194

[76] Brockhausen I, Carver JP, Schachter H. Control of glycoprotein synthesis. The use of oligosaccharide substrates and HPLC to study the sequential pathway for *N* -acetylglucosaminyltransferases I, II, III, IV, V, and VI in the biosynthesis of highly branched *N* -glycans by Hen oviduct membranes. Biochem Cell Biol. 1988;66(10):1134–51. 2975180 10.1139/o88-131

[77] Narasimhan S. Control of glycoprotein synthesis. UDP-GlcNAc:glycopeptide beta 4-N-acetylglucosaminyltransferase III, an enzyme in Hen oviduct which adds GlcNAc in beta 1–4 linkage to the beta-linked mannose of the Trimannosyl core of N-glycosyl oligosaccharides. J Biol Chem. 1982;257(17):10235–42. 6213618

[78] Bagdonaite I, Wandall HH. Global aspects of viral glycosylation. Glycobiology. 2018;28(7):443–67. 29579213 10.1093/glycob/cwy021 PMC7108637

[79] Speciale I, Laugieri ME, Noel E, Lin S, Lowary TL, Molinaro A, et al. Chlorovirus PBCV-1 protein A064R has three of the transferase activities necessary to synthesize its capsid protein N-linked glycans. Proc Natl Acad Sci. 2020;117(46):28735–42. 33139538 10.1073/pnas.2016626117 PMC7682578

[80] Poulin F, Gingras AC, Olsen H, Chevalier S, Sonenberg N. 4E-BP3, a new member of the eukaryotic initiation factor 4E-binding protein family. J Biol Chem. 1998;273(22):14002–7. 9593750 10.1074/jbc.273.22.14002

[81] Knox C, Luke GA, Blatch GL, Pesce ER. Heat shock protein 40 (Hsp40) plays a key role in the virus life cycle. Virus Res. 2011;160(1–2):15–24. 21729725 10.1016/j.virusres.2011.06.013

[82] Chan PP, Lin BY, Mak AJ, Lowe TM. tRNAscan-SE 2.0: improved detection and functional classification of transfer RNA genes. Nucleic Acids Res. 2021;49(16):9077–96. 34417604 10.1093/nar/gkab688 PMC8450103

[83] Nerenberg BTH, Taylor J, Bartee E, Gouveia K, Barry M, Früh K. The poxviral RING protein p28 is a ubiquitin ligase that targets ubiquitin to viral replication factories. J Virol. 2005;79(1):597–601. 15596852 10.1128/JVI.79.1.597-601.2005 PMC538746

[84] Bai C, Sen P, Hofmann K, Ma L, Goebl M, Harper JW, et al. SKP1 connects cell cycle regulators to the ubiquitin proteolysis machinery through a novel motif, the F-Box. Cell. 1996;86(2):263–74. 8706131 10.1016/s0092-8674(00)80098-7

[85] Iyer LM, Koonin EV, Aravind L. Novel predicted peptidases with a potential role in the ubiquitin signaling pathway. Cell Cycle. 2004;3(11):1440–50. 15483401 10.4161/cc.3.11.1206

[86] Yutin N, Wolf YI, Raoult D, Koonin EV. Eukaryotic large nucleo-cytoplasmic DNA viruses: clusters of orthologous genes and reconstruction of viral genome evolution. Virol J. 2009;6(1):223. 20017929 10.1186/1743-422X-6-223 PMC2806869

[87] Banerji S, Aurass P, Flieger A. The manifold phospholipases A of Legionella pneumophila– Identification, export, regulation, and their link to bacterial virulence. Int J Med Microbiol. 2008;298(3–4):169–81. 18178130 10.1016/j.ijmm.2007.11.004

[88] Mignery GA, Pikaard CS, Park WD. Molecular characterization of the patatin multigene family of potato. Gene. 1988;62(1):27–44. 3371664 10.1016/0378-1119(88)90577-x

[89] Colson P, De Lamballerie X, Yutin N, Asgari S, Bigot Y, Bideshi DK, et al. Megavirales, a proposed new order for eukaryotic nucleocytoplasmic large DNA viruses. Arch Virol. 2013;158(12):2517–21. 23812617 10.1007/s00705-013-1768-6 PMC4066373

[90] Cassetti MC, Merchlinsky M, Wolffe EJ, Weisberg AS, Moss B. DNA packaging mutant: repression of the vaccinia virus A32 gene results in noninfectious, DNA-Deficient, spherical, enveloped particles. J Virol. 1998;72(7):5769–80. 9621036 10.1128/jvi.72.7.5769-5780.1998 PMC110378

[91] Shenouda MM, Noyce RS, Lee SZ, Wang JL, Lin YC, Favis NA et al. The mismatched nucleotides encoded in vaccinia virus flip-and-flop hairpin telomeres serve an essential role in virion maturation. Condit RC, editor. PLOS Pathog. 2022;18(3). 10.1371/journal.ppat.1010392 PMC8956199 35290406

[92] De La Vega I, González A, Blasco R, Calvo V, Viñuela E. Nucleotide sequence and variability of the inverted terminal repetitions of African swine fever virus DNA. Virology. 1994;201(1):152–6. 8178480 10.1006/viro.1994.1277

[93] Zhang Y, Strasser P, Grabherr R, Van Etten JL. Hairpin loop structure at the termini of the Chlorella virus PBCV-1 genome. Virology. 1994;202(2):1079–82. 8030216 10.1006/viro.1994.1444

[94] Zhang W, Zhou J, Liu T, Yu Y, Pan Y, Yan S, et al. Four novel algal virus genomes discovered from Yellowstone lake metagenomes. Sci Rep. 2015;5(1):15131. 26459929 10.1038/srep15131 PMC4602308

[95] Xia Y, Cheng H, Zhong J. Hybrid sequencing resolved inverted terminal repeats in the genome of megavirus Baoshan. Front Microbiol. 2022;13:831659. 35620092 10.3389/fmicb.2022.831659 PMC9127612

[96] Wilson WH, Van Etten JL, Allen MJ. The Phycodnaviridae: The Story of How Tiny Giants Rule the World. In: Van Etten JL, editor. Lesser Known Large dsDNA Viruses. Berlin, Heidelberg: Springer Berlin Heidelberg; 2009. pp. 1–42. (Compans RW, Cooper MD, Honjo T, Koprowski H, Melchers F, Oldstone MBA, editors. Current Topics in Microbiology and Immunology; vol. 328). 10.1007/978-3-540-68618-7_1 PMC2908299 19216434

[97] DNA Secondary Structure [Internet]. VectorBuilder; [cited 2024 Nov 5]. Available from: https://en.vectorbuilder.com/tool/dna-secondary-structure.html

[98] Rux JJ, Kuser PR, Burnett RM. Structural and phylogenetic analysis of adenovirus hexons by use of High-Resolution X-Ray crystallographic, molecular modeling, and Sequence-Based methods. J Virol. 2003;77(17):9553–66. 12915569 10.1128/JVI.77.17.9553-9566.2003 PMC187380

[99] Shao Q, Agarkova IV, Noel EA, Dunigan DD, Liu Y, Wang A et al. Near-atomic, non-icosahedrally averaged structure of giant virus Paramecium bursaria chlorella virus 1. Nat Commun. 2022 Oct 29 [cited 2024 Oct 30];13. 10.1038/s41467-022-34218-4 PMC9617893 36309542

[100] Kelley LA, Mezulis S, Yates CM, Wass MN, Sternberg MJE. The Phyre2 web portal for protein modeling, prediction and analysis. Nat Protoc. 2015;10(6):845–58. 25950237 10.1038/nprot.2015.053 PMC5298202

[101] Xian Y, Avila R, Pant A, Yang Z, Xiao C. The role of tape measure protein in nucleocytoplasmic large DNA virus capsid assembly. Viral Immunol. 2021;34(1):41–8. 33074779 10.1089/vim.2020.0038 PMC8020550

[102] Zhang X, Xiang Y, Dunigan DD, Klose T, Chipman PR, Van Etten JL, et al. Three-dimensional structure and function of the *Paramecium bursaria* chlorella virus capsid. Proc Natl Acad Sci. 2011;108(36):14837–42. 21873222 10.1073/pnas.1107847108 PMC3169150

[103] Agarkova IV, Lane LC, Dunigan DD, Quispe CF, Duncan GA, Milrot E et al. Identification of a chlorovirus PBCV-1 protein involved in degrading the host cell wall during virus infection. Viruses. 2021;13(5). 10.3390/v13050782 PMC8145301 33924931

[104] Hakim M, Fass D. Cytosolic disulfide bond formation in cells infected with large nucleocytoplasmic DNA viruses. Antioxid Redox Signal. 2010;13(8):1261–71. 20136503 10.1089/ars.2010.3128

[105] Li SN, Wang JY, Yuan MJ, Yang K. Disruption of the baculovirus core gene ac78 results in decreased production of multiple nucleocapsid-enveloped occlusion-derived virions and the failure of primary infection in vivo. Virus Res. 2014;191:70–82. 25087880 10.1016/j.virusres.2014.07.019

[106] Coy SR, Gann ER, Pound HL, Short SM, Wilhelm SW. Viruses of eukaryotic algae: diversity, methods for detection, and future directions. Viruses. 2018;10(9). 10.3390/v10090487 PMC6165237 30208617

[107] Santini S, Jeudy S, Bartoli J, Poirot O, Lescot M, Abergel C, et al. Genome of *Phaeocystis globosa* virus PgV-16T highlights the common ancestry of the largest known DNA viruses infecting eukaryotes. Proc Natl Acad Sci. 2013;110(26):10800–5. 23754393 10.1073/pnas.1303251110 PMC3696832

[108] Wick RR, Filtlong. Github; 2021. Available from: https://github.com/rrwick/Filtlong?tab=readme-ov-file

[109] Wood DE, Lu J, Langmead B. Improved metagenomic analysis with kraken 2. Genome Biol. 2019;20(1). 10.1186/s13059-019-1891-0 PMC6883579 31779668

[110] Edgar RC. MUSCLE: multiple sequence alignment with high accuracy and high throughput. Nucleic Acids Res. 2004;32(5):1792–7. 15034147 10.1093/nar/gkh340 PMC390337

[111] Lee JY, Kong M, Oh J, Lim J, Chung SH, Kim JM et al. Comparative evaluation of nanopore Polishing tools for microbial genome assembly and Polishing strategies for downstream analysis. Sci Rep. 2021;11. 10.1038/s41598-021-00178-w PMC8528807 34671046

[112] Wright C, Medaka. Github; 2023. Available from: https://github.com/nanoporetech/medaka

[113] Chen S, Zhou Y, Chen Y, Gu J. Fastp: an ultra-fast all-in-one FASTQ preprocessor. Bioinformatics. 2018;34(17):i884–90. 30423086 10.1093/bioinformatics/bty560 PMC6129281

[114] Wick RR, Holt KE, Polypolish. Short-read polishing of long-read bacterial genome assemblies. Schneidman-Duhovny D, editor. PLOS Comput Biol. 2022;18(1). 10.1371/journal.pcbi.1009802 PMC8812927 35073327

[115] Quick J, Grubaugh ND, Pullan ST, Claro IM, Smith AD, Gangavarapu K, et al. Multiplex PCR method for minion and illumina sequencing of Zika and other virus genomes directly from clinical samples. Nat Protoc. 2017;12(6):1261–6. 28538739 10.1038/nprot.2017.066 PMC5902022

[116] Gouy M, Tannier E, Comte N, Parsons DP. Seaview version 5: A multiplatform software for multiple sequence alignment, molecular phylogenetic analyses, and tree reconciliation. Methods Mol Biol Clifton NJ. 2021;2231:241–60. 10.1007/978-1-0716-1036-7_15 33289897

[117] Besemer J. GeneMarkS: a self-training method for prediction of gene starts in microbial genomes. Implications for finding sequence motifs in regulatory regions. Nucleic Acids Res. 2001;29(12):2607–18. 11410670 10.1093/nar/29.12.2607 PMC55746

[118] Delcher AL, Bratke KA, Powers EC, Salzberg SL. Identifying bacterial genes and endosymbiont DNA with glimmer. Bioinformatics. 2007;23(6):673–9. 17237039 10.1093/bioinformatics/btm009 PMC2387122

[119] Hyatt D, Chen GL, LoCascio PF, Land ML, Larimer FW, Hauser LJ. Prodigal: prokaryotic gene recognition and translation initiation site identification. BMC Bioinformatics. 2010;11(1). 10.1186/1471-2105-11-119 PMC2848648 20211023

[120] Rho M, Tang H, Ye Y. FragGeneScan: predicting genes in short and error-prone reads. Nucleic Acids Res. 2010;38(20). 10.1093/nar/gkq747 PMC2978382 20805240

[121] Lazeroff M, Ryder G, Harris SL, Tsourkas PK. Phage commander, an application for rapid gene identification in bacteriophage genomes using multiple programs. PHAGE. 2021;2(4):204–13. 36147516 10.1089/phage.2020.0044 PMC9041506

[122] González-Tortuero E, Krishnamurthi R, Allison HE, Goodhead IB, James CE. Comparative analysis of gene prediction tools for viral genome annotation. 2021. Available from: http://biorxiv.org/lookup/doi/10.1101/2021.12.11.472104

[123] Salisbury A, Tsourkas PK. A method for improving the accuracy and efficiency of bacteriophage genome annotation. Int J Mol Sci. 2019;20(14). 10.3390/ijms20143391 PMC6678273 31295925

[124] Altschu SF, Gish W, Miller W, Myers EW, Lipman DJ. Basic local alignment search tool. J Mol Biol. 1990;215(3):403–10. 2231712 10.1016/S0022-2836(05)80360-2

[125] Chen IMA, Chu K, Palaniappan K, Ratner A, Huang J, Huntemann M, et al. The IMG/M data management and analysis system V.7: content updates and new features. Nucleic Acids Res. 2023;51(D1):D723–32. 36382399 10.1093/nar/gkac976 PMC9825475

[126] Paysan-Lafosse T, Blum M, Chuguransky S, Grego T, Pinto BL, Salazar GA, et al. InterPro in 2022. Nucleic Acids Res. 2023;51(D1):D418–27. 36350672 10.1093/nar/gkac993 PMC9825450

[127] Bigot T, Temmam S, Pérot P, Eloit M. RVDB-prot, a reference viral protein database and its HMM profiles. F1000Research. 2019;8:530. 32983411 10.12688/f1000research.18776.1 PMC7492780

[128] Terzian P, Olo Ndela E, Galiez C, Lossouarn J, Pérez Bucio RE, Mom R et al. PHROG: families of prokaryotic virus proteins clustered using remote homology. NAR Genomics Bioinforma. 2021;3(3). 10.1093/nargab/lqab067 PMC8341000 34377978

[129] Trgovec-Greif L, Hellinger HJ, Mainguy J, Pfundner A, Frishman D, Kiening M et al. VOGDB—Database Virus Orthologous Groups Viruses. 2024;16(8). 10.3390/v16081191 PMC11360334 39205165

[130] Zayed AA, Lücking D, Mohssen M, Cronin D, Bolduc B, Gregory AC, et al. Efam: an expanded, metaproteome-supported HMM profile database of viral protein families. Bioinformatics. 2021;37(22):4202–8. 34132786 10.1093/bioinformatics/btab451 PMC9502166

[131] Eddy SR. Accelerated profile HMM searches. Pearson WR, editor. PLoS Comput Biol. 2011;7(10). 10.1371/journal.pcbi.1002195 PMC3197634 22039361

[132] Mirdita M, Steinegger M, Söding J. MMseqs2 desktop and local web server app for fast, interactive sequence searches. Hancock J, editor. Bioinformatics. 2019;35(16):2856–8. 10.1093/bioinformatics/bty1057 PMC6691333 30615063

[133] Guindon S, Gascuel O. B Rannala editor 2003 A simple, fast, and accurate algorithm to estimate large phylogenies by maximum likelihood. Syst Biol 52 5 696–704. 14530136 10.1080/10635150390235520

