# Supplementary figures and images for: Hybrid sequencing reveals the genome of a Chrysochromulina parva virus and highlight its distinct replication strategy
Source: BMC Genomics. 2025 May 17;26:498. doi: 10.1186/s12864-025-11700-z (PMC12085832; doi:10.1186/s12864-025-11700-z)

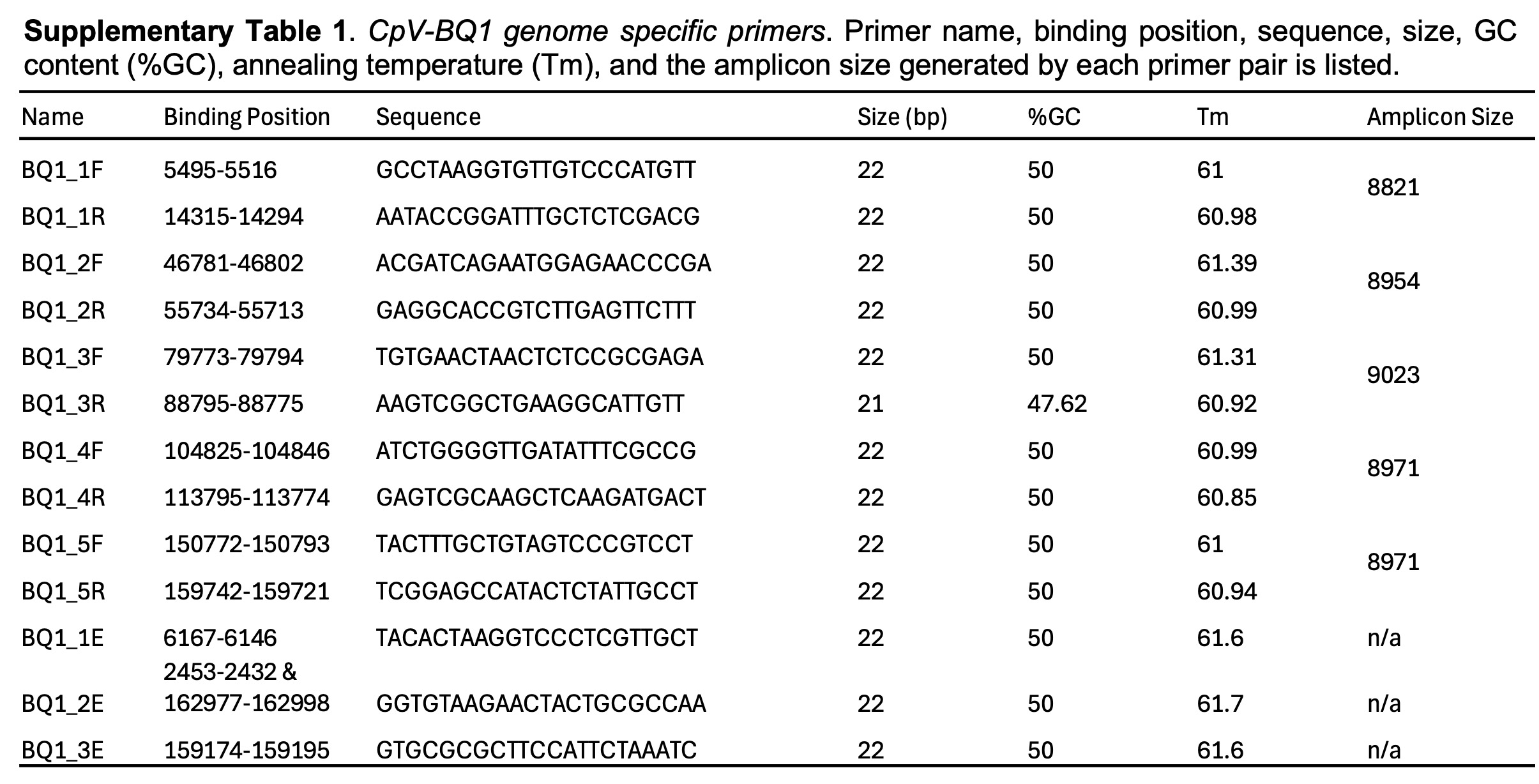

Supplement: Supplementary file 1 — Supplementary Material 1 [file 12864_2025_11700_MOESM1_ESM.jpg]

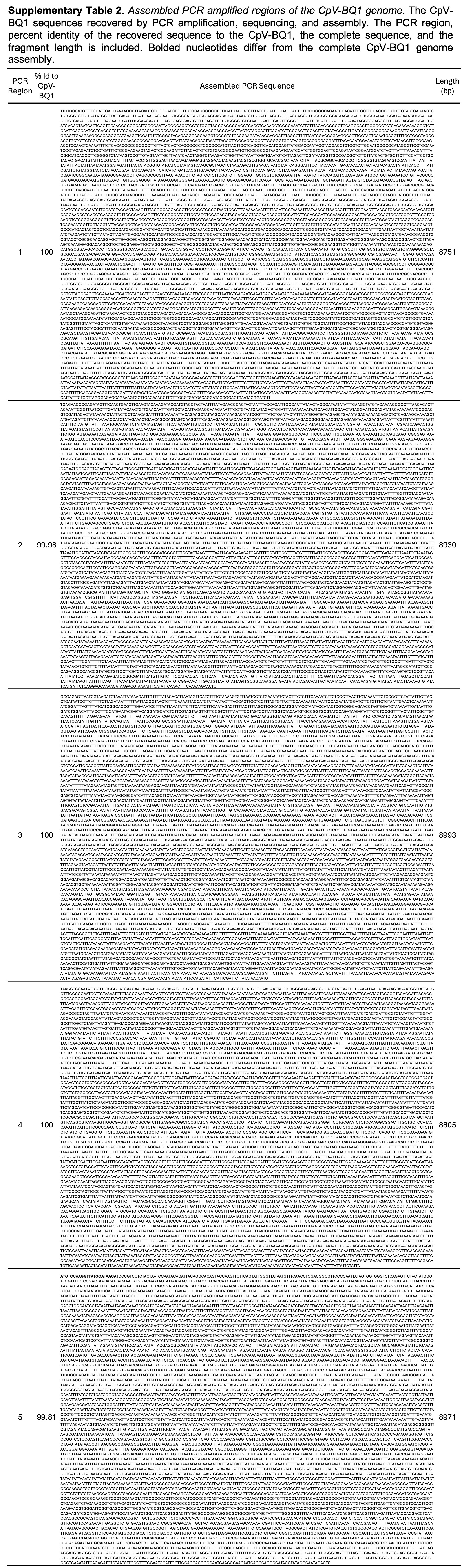

Supplement: Supplementary file 2 — Supplementary Material 2 [file 12864_2025_11700_MOESM2_ESM.jpg]

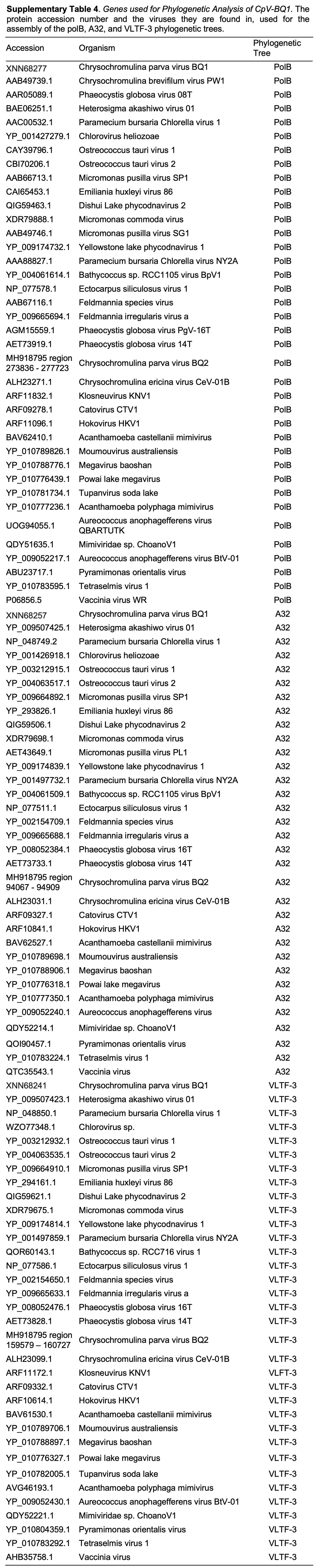

Supplement: Supplementary file 4 — Supplementary Material 4 [file 12864_2025_11700_MOESM4_ESM.jpg]
